# Global tissue transcriptomic analysis to improve genome annotation and unravel skin pigmentation in goldfish

**DOI:** 10.1038/s41598-020-80168-6

**Published:** 2021-01-19

**Authors:** Wu Gan, Yu-Wen Chung-Davidson, Zelin Chen, Shiying Song, Wenyao Cui, Wei He, Qinghua Zhang, Weiming Li, Mingyou Li, Jianfeng Ren

**Affiliations:** 1 Key Laboratory of Exploration and Utilization of Aquatic Genetic Resources, Ministry of Education, Shanghai Ocean University, Shanghai, 201306 China; 2 Department of Fisheries and Wildlife, Michigan State University, East Lansing, MI 48824 USA; 3 South China Sea Institute of Oceanology, Chinese Academy of Sciences, Guangzhou, 510301 China; 4 International Research Center for Marine Biosciences, Ministry of Science and Technology, Shanghai Ocean University, Shanghai, 201306 China

**Keywords:** Biological techniques, Genetics, Zoology

## Abstract

Goldfish is an ornamental fish with diverse phenotypes. However, the limited genomic resources of goldfish hamper our understanding of the genetic basis for its phenotypic diversity. To provide enriched genomic resources and infer possible mechanisms underlying skin pigmentation, we performed a large-scale transcriptomic sequencing on 13 adult goldfish tissues, larvae at one- and three-days post hatch, and skin tissues with four different color pigmentation. A total of 25.52 Gb and 149.80 Gb clean data were obtained using the PacBio and Illumina platforms, respectively. Onto the goldfish reference genome, we mapped 137,674 non-redundant transcripts, of which 5.54% was known isoforms and 78.53% was novel isoforms of the reference genes, and the remaining 21,926 isoforms are novel isoforms of additional new genes. Both skin-specific and color-specific transcriptomic analyses showed that several significantly enriched genes were known to be involved in melanogenesis, tyrosine metabolism, PPAR signaling pathway, folate biosynthesis metabolism and so on. Thirteen differentially expressed genes across different color skins were associated with melanogenesis and pteridine synthesis including *mitf*, *ednrb*, *mc1r*, *tyr*, *mlph* and *gch1*, and xanthophore differentiation such as *pax7*, *slc2a11* and *slc2a15*. These transcriptomic data revealed pathways involved in goldfish pigmentation and improved the gene annotation of the reference genome.

## Introduction

Goldfish (*Carassius auratus auratus*) is an important ornamental fish, and has been subjected to extremely intensive artificial selection during its domestication history^1,2^. Goldfish breeding occurred in the Song dynasty of China over 1000 years ago^1^. At least 180 variants and 70 genetically established strains are currently generated according to their body shape, coloration, scale, as well as fin, eye, and hood morphology^1,3^. Such wide diversity of phenotypes makes goldfish an excellent model to study vertebrate development, evolution and human disease^2,3^.

Goldfish was domesticated from wild grey crucian carp (*C. auratus*)^1^. The red color skin was the first trait that was fixed and distinct from the ancestral trait^1^. Skin color plays key roles in social interaction, including species communication, predation avoidance, and camouflage in teleosts^4^. Coloration results from pigments synthesized by chromatophores^5^. Compared to mammals that only contain melanocytes, fishes possess more than five types of chromatophores^6,7^. The genetic basis of chromatophore development and differentiation is conserved from teleosts to mammals^5^. Thus, teleost fishes are useful models to define genetic mechanisms of pigmentation. Compared to tetrapods, fishes underwent teleost-specific whole genome duplication (WGD) which contributes to pigment diversification^8^. Specially, the common ancestor of goldfish and common carp underwent one more round of WGD occurred about 8–14.4 million years ago^9,10^, and enabled further increase in the complexity of pigmentation gene regulations.

In goldfish, the unparalleled features of phenotypic diversity and extra round of WGD provide ample opportunities to examine the relationship between phenotypes and genotypes. Recently, a high quality reference genome for a common goldfish strain, *Wakin*, has been assembled^10^. However, the gene models of this assembly were predicted with limited RNA-Seq data. Additional data on alternative isoforms and untranslated regions are critical for understanding the phenotypic diversity in goldfish. In this study, we combined the PacBio and Illumina sequencing to obtain a comprehensive transcriptomic landscape of goldfish stain, *Oranda*. PacBio Single-molecule real-time (SMRT) long read sequencing displays superiority in higher complete readouts of full-length transcripts and greater accuracy in splice isoforms over the Illumina short-read sequencing^11^. It has been widely applied to obtain full-length transcripts in species with reference genomes such as human^12^, zebrafish^13^, rice^14^, and sorghum^15^, and species without reference genomes such as *Gymnocypris selincuoensis*^16^ and *Misgurnus anguillicaudatus*^17^.

We combined the PacBio long-read sequencing and Illumina short-read sequencing technologies to obtain a more complete transcriptome in goldfish. The goldfish genome annotations were further improved with these transcriptome data and novel genes and different isoforms were identified compared with the reference annotation. In addition, we also identified genes involved in pigmentation pathways using goldfish with different color skin. The transcriptomic data and genomic resources provided in this study improved the goldfish genome annotation and enhanced the understanding of pigmentation pathways and would facilitate future researches on genetic basis of diverse phenotypes.

## Results

### PacBio full-length sequencing and processing

**Figure 1:**
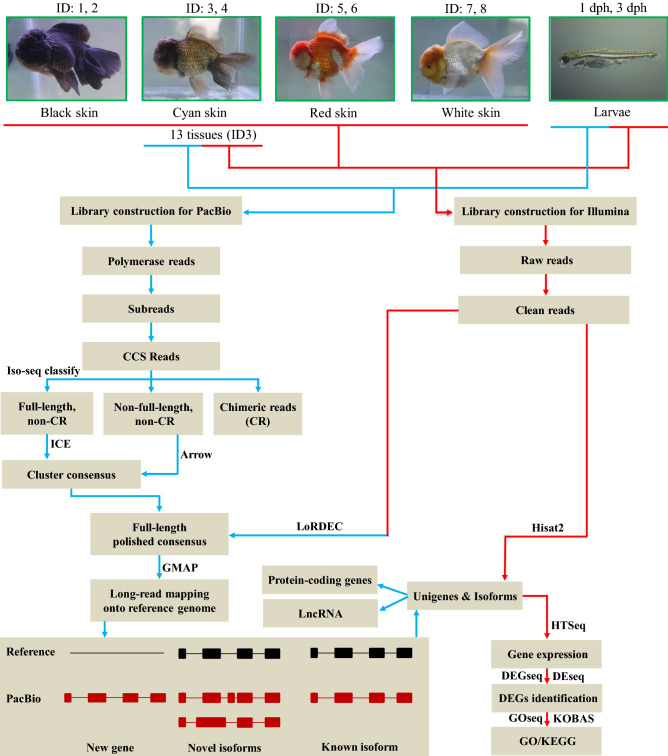
Overview of the experimental design and the data processing pipeline. This study consisted of experimental design, library construction and sequencing, and data processing and analyses. Goldfish samples include 13 tissues from one cyan fish, four color skins and larvae at 1 and 3 dph. All 22 samples were subject to Illumina sequencing, and only 13 tissues from one cyan fish and larvae at 1 and 3 dph were used for PacBio sequencing. The figures were constructed in Microsoft Office PowerPoint.

To ensure higher coverage of genes and their isoforms, we pooled RNA samples from 13 tissues of one cyan-skin adult goldfish and larvae at one- and three-days post hatch (dph) for PacBio sequencing (Fig. 1). We obtained 25.52 Gb post-filter polymerase reads that contained 11,448,193 subreads with an average length of 2,184 bp and N50 length of 3,108 bp, respectively (Table 1). The subreads were utilized to generate circular consensus sequences (CCS) and a total of 632,099 CCS reads were obtained. Among the CCS reads, 573,366 (90.71%) reads were identified as full-length non-chimeric (Flnc) transcripts (containing 5′ primer, 3′ primer and the polyA tail), 47,515 (7.52%) reads were identified as non-full length non-chimeric (Nflnc) reads, and 11,218 (1.77%) were identified as chimeric reads (CR). The mean length and N50 length of Flnc reads were 2920 bp and 3924 bp, respectively (Table 1). Subsequently, the Flnc reads were clustered by Iterative Clustering for Error Correction (ICE) algorithm to generate the consensus sequences and the Nflnc sequences were used to polish the ICE consensus sequences. A total of 300,733 full-length polished consensus sequences were obtained with an average length of 2949 bp and N50 length of 4000 bp. The polished consensus sequences were further rectified with Illumina RNA-Seq data, resulting in a slightly improved average length of 2956 bp and N50 length of 4017 bp (Table 1).

**Table 1:** Statistical information of PacBio Iso-Seq sequencing and processing.

	Polymerase	Subread	CCS	Flnc	Consensus sequences	
					ICE clustered and Nflnc polished	Illumina polished
Number	1,379,351	11,448,193	632,099	573,366	300,733	300,733
Mean Length (bp)	18,500	2183	3007	2920	2949	2956
N50 Length (bp)	–	3108	3927	3924	4000	4017

### PacBio data genome mapping

**Figure 2:**
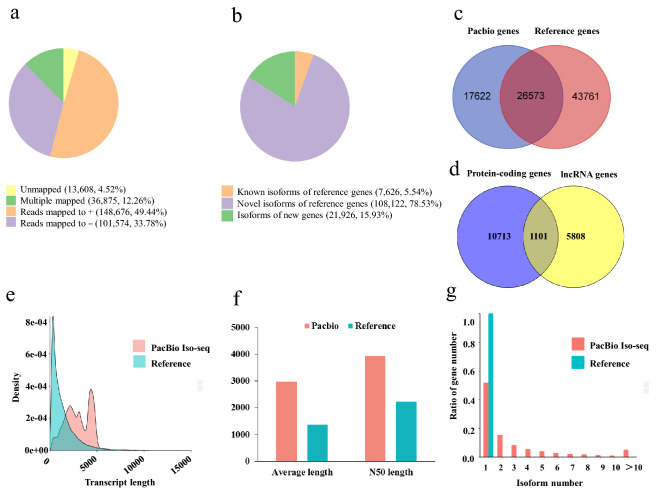
GMAP mapping result and the comparison of goldfish transcript isoforms between the PacBio Iso-Seq and reference genome annotation. (**a**) Statistics of the consensus sequences mapped onto the reference genome. (**b**) Classification of transcript isoforms corresponding to the reference genome. (**c**) The comparison of gene numbers between Pacbio Iso-Seq and the reference genome annotation. (**d**) Classifications of novel gene loci identified in PacBio Iso-Seq. (**e**) The comparison of transcript length distribution between Pacbio Iso-Seq and the reference genome annotation. (**f**) Comparisons of the average length and N50 length of transcripts between Pacbio Iso-Seq and the reference genome annotation. (**g**) The comparison of transcript isoform number between Pacbio Iso-Seq and the reference genome annotation. The figures were constructed in ggplot2 (https://ggplot2.tidyverse.org).

The corrected consensus sequences were mapped against the goldfish reference genome to further improve gene structure annotation using GMAP. More than 95.48% sequences (287,125) were successfully mapped onto the reference genome while the remaining 13,608 (4.52%) sequences were unmapped. Among the mapped sequences, 250,250 (83.21%) sequences were uniquely mapped, and of which, 148,676 (49.44%) sequences were mapped onto the positive strand while 101,574 (33.78%) sequences were mapped onto the negative strand of the reference genome. The remaining 36,875 (12.26%) sequences showed multiple alignments on the reference genome (Fig. 2a).

### Isoform detection and characterization

A total of 137,674 non-redundant transcript isoforms were obtained and classified into three types compared to reference genome annotation (Fig. 2b). The first type, or the known isoforms of reference genes, contained 7,626 isoforms accounted for 5.54% of the identified isoforms. The second type, novel isoforms of reference genes, consisted of 108,122 isoforms accounted for most of the identified isoforms (78.53%) (Supplementary Table S1). The third type, isoforms of new genes, which were aligned to the un-annotated regions of the reference genome, comprised 21,926 isoforms accounted for 15.93% of the identified isoforms.

The non-redundant isoforms were aligned to 44,195 gene loci, of which 26,573 (60.13%) gene loci were overlapped with reference protein-coding genes, and the remaining 17,622 (39.87%) gene loci were aligned to un-annotated loci and identified as new genes compared to the reference genome annotation (Fig. 2c, Supplementary Table S2). These new gene loci contained both protein-coding genes (10,713 loci) and nonprotein-coding genes (5,808 loci), namely, lncRNA genes. In addition, 1,101 loci contained both protein-coding genes and lncRNA genes. In total, 11,814 gene loci contained protein-coding genes (Fig. 2d).The distribution of isoform length was compared between PacBio Iso-Seq transcripts and the reference genes. The density distribution curve showed that PacBio Iso-Seq isoforms were longer than the reference genes (Fig. 2e). The average length and N50 length of PacBio Iso-Seq isoforms were 2985 bp and 3939 bp, respectively, whereas those of reference genes were 1378 bp and 2227 bp, respectively (Fig. 2f). Moreover, 21,291 out of 44,195 (48.18%) PacBio Iso-Seq genes possessed two or more transcript isoforms with an average of 3.12 isoforms per gene while each reference gene was only annotated with a single isoform (Fig. 2g, Supplementary Table S3).

### Annotation analysis of new transcripts and genes

The 13,608 unmapped transcripts (Fig. 2a) and 17,622 new genes (Fig. 2c) were annotated with seven databases including NR, NT, Pfam, KOG, SwissProt, KEGG and GO databases. For the unmapped transcripts, 3868 transcripts were annotated in all seven databases and 13,460 genes were annotated at least in one database. For the new genes, 2446 genes were annotated in all seven databases and 17,493 genes were annotated at least in one database (Supplementary Fig. S1, Supplementary Table S4). We further analyzed these new genes after excluding 5808 lncRNA genes (Fig. 2d). Among the remaining 11,814 protein-coding genes, 4431 genes were annotated with both NCBI-nr and Swiss-protein database. These protein-coding genes were compared with those annotated in goldfish genome on Ensembl database (version 101.1). The results showed that 591 protein-coding genes overlapped with the Ensembl annotation. Therefore, these 4431 loci most likely encoded reliable protein-coding genes.

### Alternative splicing analysis

**Figure 3:**
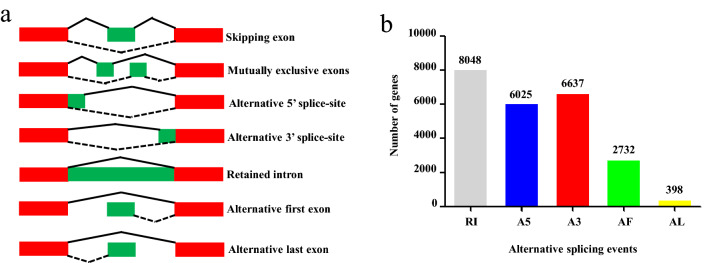
Alternative splicing (AS) with different models. (**a**) Seven types of AS. (**b**) The number of AS events occurred in detected genes from SMRT sequencing. The figures were constructed in Microsoft Office PowerPoint and Excel.

Alternative splicing (AS) formation could be classified into seven models (Fig. 3a): SE (skipping exon), MX (mutually exclusive exon), A5 (alternative 5′ splice site), A3 (alternative 3′ splice site), RI (retained intron), AF (alternative first exon) and AL (alternative last exon). In the PacBio Iso-Seq data set, we found that 15,380 genes underwent 59,014 AS events (Fig. 3b, Supplementary Table S5). The major AS type was RI, accounted for 17,593 AS events. Interestingly, MBNL1 (Gene ID: CA00019485) underwent the most AS events. MBNL1 encodes the muscle blind proteins which are one of the C3H-type zinc finger proteins^18^. This gene regulates programmed alternative splicing from development to diseases^19^. In summary, the genes encoded in goldfish genome had complex AS events.

### Classification of non-coding RNAs

**Figure 4:**
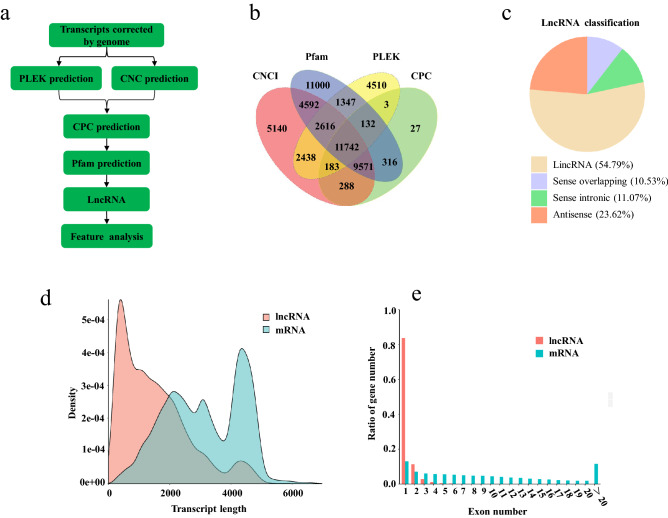
LncRNA identification and feature analysis. (**a**) The workflow of lncRNA identification. (**b**) The number of lncRNAs identified by CNCI, CPC, Pfam and PLEK methods. (**c**) The percentage of four classes of lncRNAs. (**d**) The comparison of transcript length distribution between lncRNA and mRNA. (**e**) The comparison of exon number between lncRNA and mRNA. The figures were constructed in ggplot2 (https://ggplot2.tidyverse.org).

A total of 11,742 lncRNA transcripts encoded in 10,240 gene loci (Supplementary Table S6) were identified (Fig. 4b). The lncRNAs were classified into four types according to their positions on the reference genome. Large intergenic noncoding RNA (lincRNA) accounted for the majority of lncRNA (54.79%), whereas antisense lncRNA accounted for 23.62% of the lncRNA. In addition, sense intronic and sense overlapping lncRNAs accounted for 11.07% and 10.53% of the total lncRNAs, respectively (Fig. 4c). The length distribution curves of lncRNA and mRNA transcripts showed that lncRNA was shorter than mRNA (Fig. 4d). Moreover, 83.7% (9,826) lncRNA genes possessed only one exon while mRNA genes exhibited an average of 9.9 exons (Fig. 4e). These lncRNA transcripts were compared with 2,806 lncRNA genes annotated in goldfish genome on Ensembl database (version 101.1). The results showed that 433 lncRNAs were overlapped between our prediction and the Ensembl annotation.

### Global tissue transcriptomic analysis

**Figure 5:**
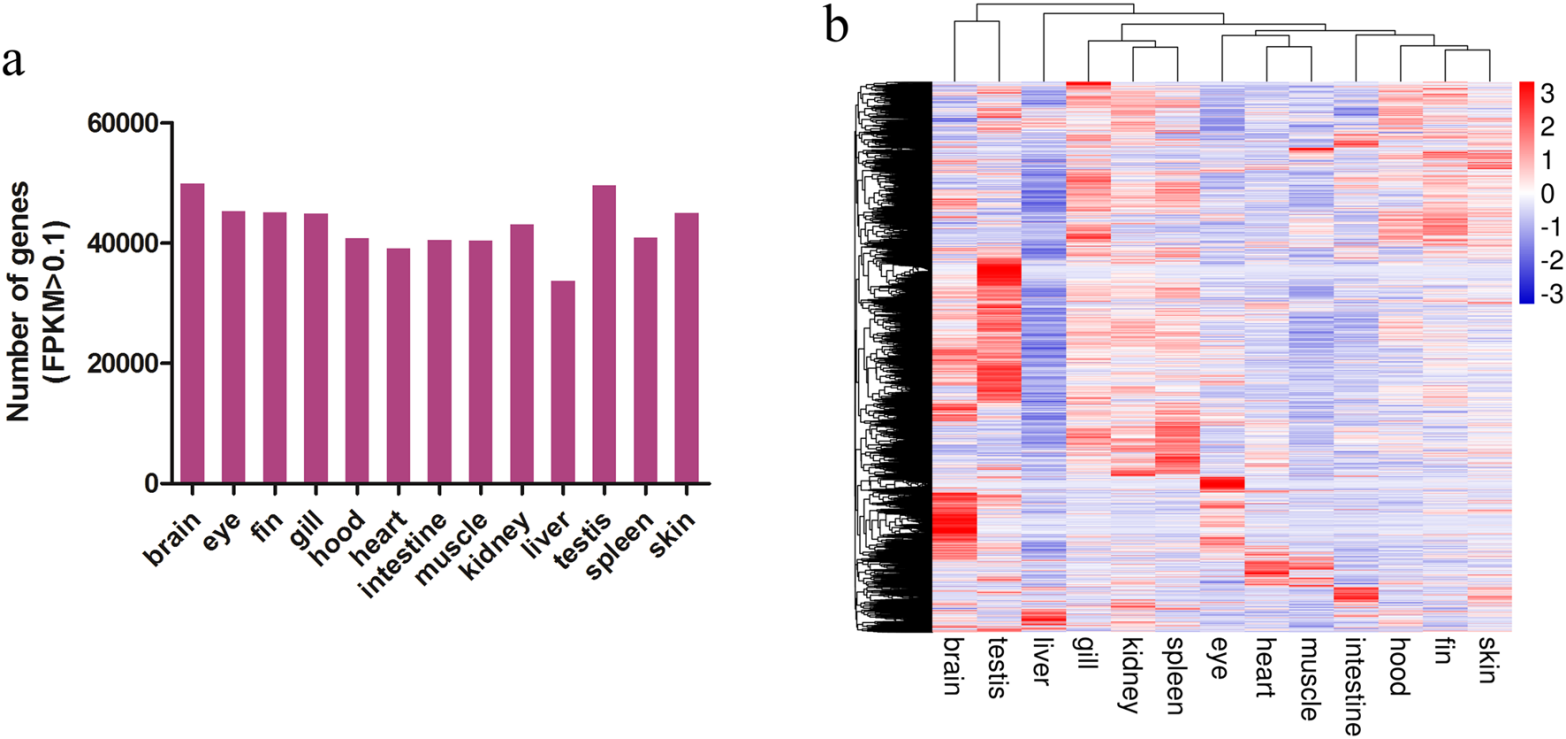
Gene expression profiles of 13 adult goldfish tissues. (**a**) The number of expressed genes (FPKM > 0.1) in each tissue. (**b**) Heatmap of gene expression levels in each tissue. The figures were constructed in Microsoft Office Excel and pheatmap R package 1.0.12 (https://CRAN.R-project.org/package=pheatmap).

The Illumina clean reads were mapped onto the goldfish reference genome, and the expression level of each isoform was estimated using the updated GFF file containing 87,946 genes from this study and the reference genome. Each Illumina RNA-Seq libraries generated more than 5.75 Gb clean data. The mapping rate of clean reads varied from 67.09% (Sn1) to 86.56% (dph1) (Supplementary Table S7). Around five thousand genes were expressed (FPKM >  = 0.1) in each adult tissue, with the brain and testis tissue expressed the most genes (59,789 and 59,286) (Fig. 5a). Gene expression cluster analysis was performed using pheatmap R package 1.0.12 with hierarchical clustering method of Euclidean distance. The heatmap results showed that tissues clustered into several groups. The skin, fin, hood, and intestine were clustered together while muscle, heart and eyes formed the second group. The spleen, kidney and gill clustered into the third group. The testis and brain were clustered together probably due to the number of closely expressed genes. In general, these clustering patterns demonstrated that functionally related tissues shared similar gene expression profiles (Fig. 5b).

### Skin- and color-specific transcriptomic analysis

**Figure 6:**
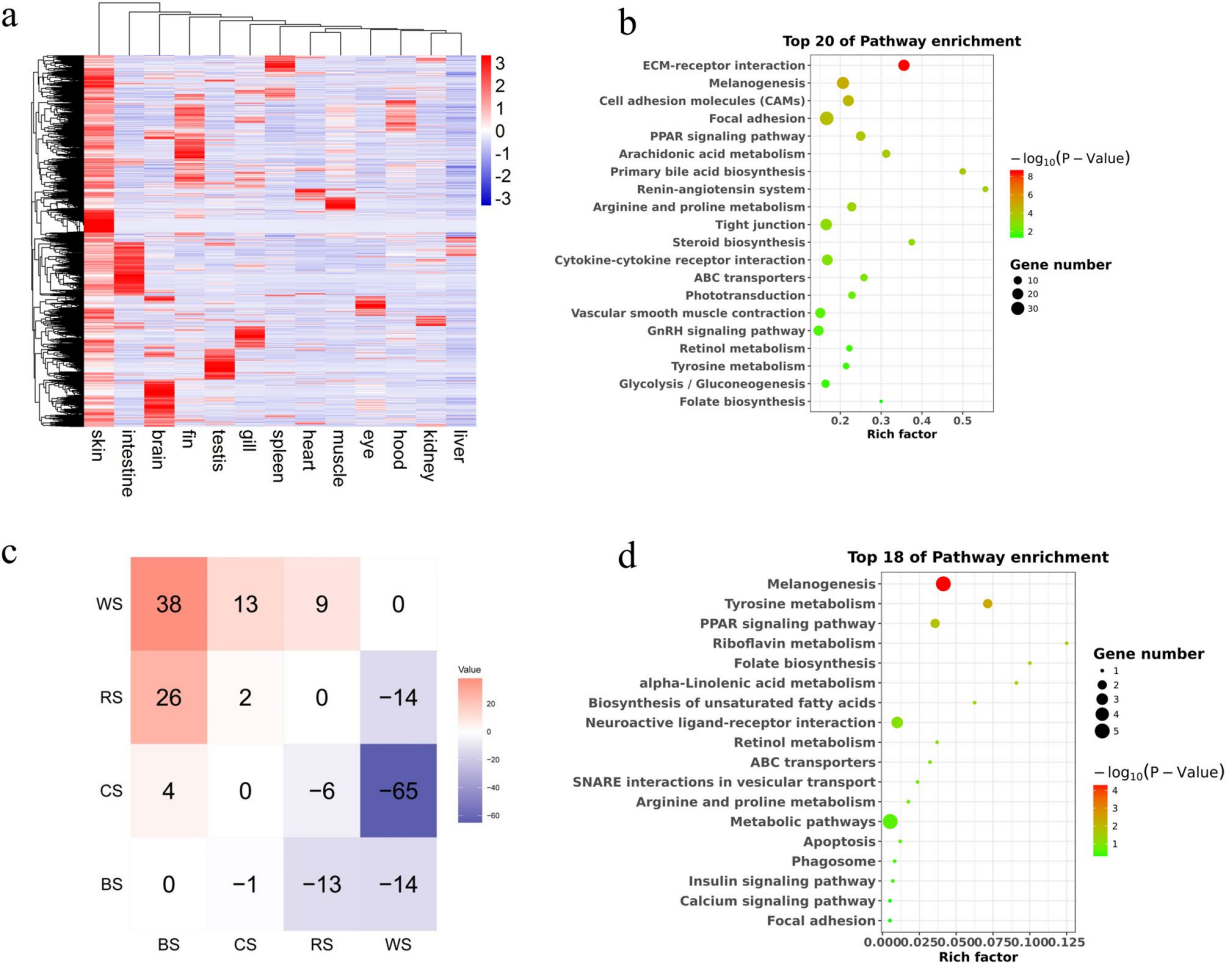
Analyses of skin- and color-specific gene expressions. (**a**) Heatmaps of skin-specific gene expression. (**b**) Top 20 pathways enriched with skin-specific genes. (**c**) Comparisons of differentially expressed genes between four different color skins. (**d**) Top 18 pathways enriched with differentially expressed genes in different color skins. BS: black skin; CS: cyan skin; RS: red skin; WS: white skin. The figures were constructed in pheatmap R package 1.0.12 (https://CRAN.R-project.org/package=pheatmap) and ggplot2 (https://ggplot2.tidyverse.org).

To identify skin-specific genes, differentially expressed genes were analyzed between skin and other tissues. The genes up-regulated in skin (fold change > 2 and FDR < 0.05) compared with at least other 9 tissues were defined as the skin-specific expressed genes (Fig. 6a). KEGG pathway analysis revealed that the skin-specific genes were significantly enriched in several signaling pathways, including ECM-receptor interaction, melanogenesis, cell adhesion molecules, focal adhesion, tyrosine metabolism, and so on (Fig. 6b).

To further identify the genes and pathways related to color-specific skin, we investigated gene expression profiles of four different color skins (black, cyan, red, and white) with RNA-Seq. A total of 162 DEGs were identified among four color skins (Fig. 6c, Supplementary Table S8). The DEGs of color-specific skin were significantly involved in melanogenesis, tyrosine metabolism, riboflavin metabolism, and folate biosynthesis (Fig. 6d). We found that 13 out of the 162 DEGs were involved in pigmentation pathways^8,20^ (Supplementary Table S9). Generally, these 13 DEGs showed low expression levels in white skin. Mitf and Ednrb were involved in melanophore development. Mc1r and Mc5r were associated with the regulation of melanogenesis. Tyr and Tyrp1b were components of melanosomes. Mlph were important for melanosome transport. All of them played a role in the biological processes of melanogenesis^5,8^. On the other hand, Gch1 was involved in pteridine synthetic pathway. Pax7, Slc2a11, and Slc2a15 were associated with xanthophore development and differentiation^8,21^.

**Figure 7:**
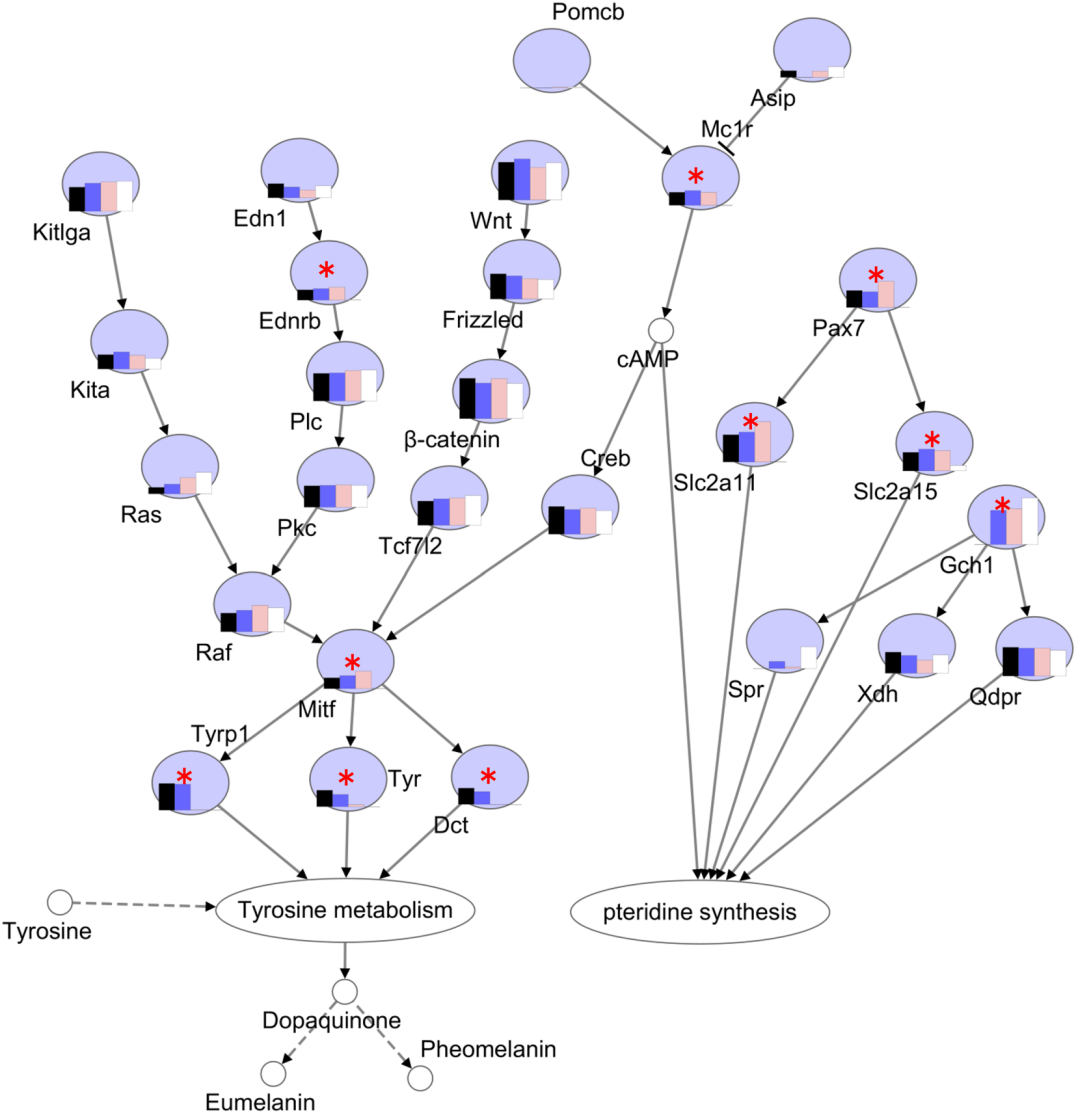
The differentially expressed genes in the gene regulatory network of pigment synthesis. * indicates differentially expressed genes between four different color skins. Four color bars in each circle represent the relative expression level of each gene in four color skins. Solid arrow represents direct positive regulation, “T” shape solid line represents direct negative regulation, and dotted arrow represents indirect positive regulation. The figures were constructed in Microsoft Office PowerPoint.

To gain further insight of the pigmentation regulation in different color skins, we used the expression levels of the pigmentation genes from four-color skins and constructed a gene regulatory network of pigmentation in goldfish (Fig. 7). As expected, the network of melanogenesis, tyrosine metabolism, and pteridine synthesis interacted with each other, and mediated by at the secondary message cAMP .

## Discussion

### High-quality Iso-Seq significantly improved goldfish genome annotation

Long-read sequencing technology can capture full-length transcripts without assembly and overcome the difficulties presented by short-reads^22^. However, PacBio Iso-Seq is still rarely used in aquaculture animals. In the present study, combining the PacBio Iso-Seq and Illumina RNA-Seq, we firstly reported the high coverage of full-length transcriptome that significantly improved the published goldfish genome annotation.

The Iso-Seq data from 13 pooled samples generated 300,733 high-quality sequences. The mean length and N50 length of the full-length transcripts were 2956 bp and 4,017 bp, which were longer than those of the de novo assembled transcripts generated with RNA-Seq in goldfish (879.5 and 1318 bp), and in other fish species (815 and 1479 bp)^23–25^. Moreover, our results are comparable to those generated with PacBio Iso-Seq in *Gymnocypris selincuoensis* (3,509 and 3,870 bp)^16^. In addition, the PacBio Iso-Seq technology exhibits the advantage of discovering more novel isoforms and AS events in many species, including reference genome-free species^26^. In this study, we identified 137,674 isoforms from 44,195 gene loci, including 108,122 (78.53% of total isoforms) novel isoforms of previously known genes and 17,622 novel genes. Even in well annotated genome of model animal, more novel isoforms could still be identified using PacBio Iso-Seq. A recent study on zebrafish using PacBio Iso-Seq discovered 1835 novel isoforms of known genes that have not been annotated in the RefSeq catalog^13^. Compared to goldfish, *Gymnocypris selincuoensis*, the cyprinid fish living in lake Selincuo on the Qinghai-Tibet Plateau, may present fewer AS events (2069 pairs) in full-length transcripts^16^. Zhang et al. revealed 1164 full-length transcripts underwent AS events via long-read sequencing in Pacific white shrimp *Litopenaeus vannamei*^27^.

We investigated the gene expression pattern of the newly discovered 11,814 protein-coding genes (Fig. 2d). The heatmap showed that most of the genes were specifically and dominantly expressed in one or two functionally similar tissues examined (Supplementary Table S10, and Fig. S2). We also investigated the gene expression pattern of the 11,742 lncRNA transcripts. Similar to the novel protein-coding genes, the lncRNA genes were also specifically and dominantly expressed in one or two functionally similar tissues (Supplementary Table S11, and Fig. S3). This is the possible reason why these newly identified protein-coding genes were missing in the reference genome annotation with limited RNA-Seq data.

To evaluate the gene completeness of the transcriptome, the 137,674 non-redundant transcript isoforms were analyzed and assessed by Benchmarking Universal Single-Copy Orthologs (BUSCOs)^28^ using the vertebrate core gene sets (vertebrata_odb10), resulting in 2,673 complete (79.7%), 145 fragmented (4.3%), and 536 (16.0%) missing BUSCOs of total 3,354 BUSCOs (Supplementary Fig. S4). Higher percentage of the missing BUSCOs than the reference genome annotation (5% missing) is probably due to low coverage of the goldfish transcriptome by PacBio Iso-Seq sequencing (the polymerase reads number is about tenfold of total genes, Supplementary Table S12). This is also why many reference genes were not covered by PacBio Iso-Seq genes (Fig. 2C). Combined with the reference genome annotation, our work greatly improved goldfish genome annotation, identified more protein-coding genes and more isoforms for each gene.

### Identification of color-specific genes

Skin color in teleost fish is essential for social events, including avoiding predators, catching prey and communicating with conspecifics^4^. Coloration is represented by the interaction of chromatophores, including melanocytes, xanthophores, erythrophores, and cyanophores^5^. Emerging studies have revealed the genetic mechanism of skin pigmentation in fish. For instance, both blue and brown colors are developed from the implicit homozygous, the former is controlled by one pair and the latter is controlled by four pairs of Mendelian factors^29^. Whole genomic sequencing of goldfish with various body color phenotypes demonstrated that the presence of two evolutionally-asymmetric subgenomes in the goldfish genome may contribute to production of a higher number of varieties in body colors compared to other teleost fish^30^. In this study, we identified color-specific genes and revealed that DEGs of color-specific skin were significantly involved in melanogenesis, tyrosine metabolism, riboflavin metabolism, and folate biosynthesis. In accordant with goldfish, in *Triplophysa siluroides,* the candidate genes related to skin color variation mainly participate in melanosome, melanin metabolic process, and melanin-concentrating hormone activity^31^. Moreover, the differently expressed genes in different colored skin of koi carp have been shown to be enriched in melanogenesis and tyrosine metabolism^32^. Tyrosine metabolism genes are also involved in the gray-to-red body color formation of red crucian carp^33^.

Due to diverse color pattern, goldfish could be a perfect model to study the genetic mechanism of pigmentation. The red-colored goldfish was used for studying the neuroendocrine effects on body color, because it contains iridophores and xanthophores but not melanophores in the scales or skin^34^. Transcriptome sequencing of red crucian carp and white crucian carp identified that *mitfa*, *tyr*, and *tyrp1* were significantly down-regulated and *gch1* was up-regulated in red crucian carp^35^. In this study, we compared transcriptomes of four goldfish skins with different colors and obtained 162 DEGs. Among the DEGs, *mitf*, *tyr*, *tyrp1*, and *ednrb* played important roles during the melanocyte development^20^. Mitf is a microphthalmia-associated transcription factor, which also regulates the development of melanocyte in mammals, and determines the formation of melanocytes and iridophores in zebrafish^36^. After FSGD, *mitf* have been duplicated in teleost fishes. For instant, zebrafish genome encoded two paralogous genes of *mitf*, namely *mifta* and *miftb. Mitfa* is involved in development of chromatophore, while *mitfb* is expressed in retinal pigment epithelium^37^. The melanocortin receptors (MCRs), belonging to G-protein coupled receptors (GPCRs), exert multiple functions in the control of pigmentation in the melanocortin system^38^. Five types of melanocortin receptors (MC1R, MC2R, MC3R, MC4R, and MC5R) have been identified in goldfish^39^, and *mc1r* and *mc5r* were expressed in the xanthophores in scales^34^. In this study, *mc1r* and *mc5r* had different expression level among the skins and had no expression in the white skin. GTP cyclohydrolase I (Gch1), the rate-limiting enzyme in pteridine synthesis, catalyzes the de novo synthesis of tetrahydrobiopterin (H4biopterin) from GTP^5^. Gch1 is also an essential cofactor for all nitric oxide synthases, and protects skin cells by restoring cellular H4biopterin and NO against radiation-induced damage^40^. *GchI* expression is an initial step for melanophore and xanthophore differentiation due to its involvement in different component pathways^5,41^. The DEG *gch1* had high expression in three skins, which suggested that *gch1* maybe play an important role in goldfish pigmentation. Three other DEGs, *pax7a*, *slc2a11*, and *slc2a15*, may have functions in xanthophore differentiation in goldfish as previous study showed that Pax7a, Slc2a11 and Slc2a15 are important for the development and differentiation of xanthophore and leucophore in medaka^21^.

In addition to protein-coding genes, lncRNAs may also be involved in the skin color variation^32,42^. We investigated lncRNA expression across four color skins (Supplementary Table S13 and Fig. S5) and identified the 140 differentially expressed lncRNAs between the different color skins (Supplementary Table S14 and Fig. S6, 7). Further investigation is required to confirm their functions in skin color variation.

### Comparison of ohnolog genes located in two subgenomes

The common ancestor of goldfish and common carp underwent a fourth round of WGD (Cs4R, carp-specific WGD) approximately 8–14.4 million years ago^9,10^. Cs4R was suggested to be an allotetraploidization event, which is the doubling of a complete set of chromosomes following interspecific hybridization of diploid progenitors (2n = 50)^43^. According to the distribution of transposable elements on the goldfish chromosomes (2n = 100), Kon et al. partitioned the goldfish chromosomes into two subgenomes: L (relatively long) and S (relatively short)^30^. We compared the number of isoforms, exon phases, isoform expression variance and entropy between the 5,404 pairs of ohnolog genes on L and S subgenomes (The ohnolog pairs kindly provided by Prof. Yoshihiro Omori). The genes located in the L subgenome (L-genes) had slightly more isoform number (pairwise t test *p* = 0.001081, Supplementary Table 15.1 and higher FPKM expression (pairwise t test *p* = 0.001, Supplementary Table 15.2 than the genes located in the S subgenome (S-genes), but the tissue entropy (or tissue specificity) of the L-genes was slightly lower (higher) than that of the S-genes (p = 0.066 (0.030)) (Supplementary Table 15.3, 15.4). The isoform entropy and standard deviation of expression of the L-genes were larger than the S-genes in most tissues (Supplementary Table 15.5, 15.6, Supplementary Fig. S8), suggesting the L-genes expressed more diversely across different isoforms. There was no significant difference of isoform specificity between L and S sub-genome, although the isoform specificity was lower on the L-genome (Supplementary Fig. S9). In summary, genes located in the L and S subgenomes evolved asymmetrically^30^, and that the L subgenome is the dominant subgenome, which is more often preserved and similar to the ancestral state^44,45^.

## Conclusion

In summary, our work demonstrated that full-length transcriptome sequencing has advantages in identifying novel isoforms, new gene loci and AS events. Our work significantly improved goldfish genome annotation. In addition, we presented pigment genes expression profile from different color goldfish skins, and this work enhanced our understanding of the pigmentation regulatory pathways in goldfish.

## Materials and methods

### Goldfish source and tissue collection

Eight one-year-old *Oranda* goldfish (body weight: 225 ± 54 g, body length: 11.8 ± 0.5 cm; mean ± S.E.M.) were kindly provided by Qingyuan Goldfish Tang Co., LTD (Qingyuan, Guangdong, China). These fish were transported to and maintained on the Campus of Shanghai Ocean University (Lin’gang, Shanghai, China). Fish were anesthetized with tricaine methanesulfonate (MS-222). Thirteen tissues, including the hood, brain, eye, gill, pectoral fin, skin, muscle, heart, liver, spleen, intestine, kidney and testis, were dissected from one cyan-skin fish (Sample ID: 3) (Fig. 1). Skin tissues were collected from eight fish with different colors including two black (ID: 1, 2), two cyan (ID: 3, 4), two red (ID: 5, 6) and two white fish (ID: 7, 8). Five *Ranchu* goldfish larvae at one and three dph were also collected (Fig. 1).

### RNA isolation, full-length cDNA library construction and PacBio sequencing

Total RNA was isolated from each sample using TRIzol reagent according to the manufacturer’s manual (Thermo Fisher Scientific, USA). The total RNA samples were pooled equally from 13 tissues of one cyan-skin adult goldfish, and *Ranchu* larvae at one and three dph, and purified using oligo-dT magnetic beads. PolyA RNA was then reversely transcribed into cDNA using the Clontech SMARTer PCR cDNA Synthesis Kit (TaKaRa Bio, Japan). Four fractions with different insert size (1–2 kb, 2–3 kb, 3–6 kb and 0.5–6 kb) were selected using the BluePippin system (Sage Science, Inc., USA). The resulting cDNAs were amplified by KAPA HiFi PCR Kits (Kapa Biosystems, Inc., USA). SMRTbell libraries were constructed using SMRTbell Template Prep Kit 1.0 (PacBio, USA). Twelve SMRT cells were used to sequence SMRTbell libraries (nine cells for the mixed libraries of 1–2 kb, 2–3 kb, 3–6 kb and three cells for the 0.5–6 kb library) on the PacBio Sequel system (Supplementary Table S12).

### Illumina short-read library construction and sequencing

Sequencing library for each sample was constructed using a TruSeq RNA Library Preparation Kit (Illumina, USA) following the manufacturer’s protocols. In brief, mRNA from each sample was purified from total RNA utilizing oligo-dT magnetic beads and fragmented with divalent cations. First strand cDNA was synthesized utilizing with M-MuLV Reverse Transcriptase (RNase H-) and random hexamer primers. Subsequently, DNA polymerase I, and RNase H were used to synthesize second cDNA strand. Through DNA end repair, a single nucleotide A was added at the 3′-end, and the DNA fragments were ligated to Illumina adaptors. To isolate DNA fragments of preferential ~ 300 bp in length, the library fragments were purified with AMPure XP system (Beckman Coulter, USA). PCR was then amplified using Phusion High-Fidelity DNA polymerase with Universal PCR primers and Index (X) Primers. Finally, the PCR fragments were purified with AMPure XP system again, and each library quality was assessed with the Agilent Bioanalyzer 2100 system (Agilent Technologies, Inc. USA)^46,47^.

The clustering for the index-coded samples was carried on a cBot Cluster Generation System by using the TruSeq PE Cluster Kit v3-cBot-HS (Illumina) according to the manufacturer’s specifications. After cluster generation, the libraries were sequenced with the paired-end 150 bp reads model on an Illumina HiSeq X Ten platform^46,47^.

### PacBio data filtering and processing

PacBio Iso-Seq raw reads were classified and clustered into transcript consensus using the PacBio SMRT Link v5.1 pipeline (Fig. 1). After data filtering, including adapter removal and elimination of low-quality regions, the subreads were utilized to generate CCS (parameters: –minPasses = 2, minPredicted accuracy = 0.8). The Iso-Seq classifying tool categorized the CCS into full-length non-chimeric (Flnc), non-full length non-chimeric (Nflnc) and chimeric reads (CR), based on cDNA primers and poly-A tail signal. Subsequently, the Flnc reads were clustered by Iterative Clustering for Error Correction (ICE) algorithm to generate the cluster consensus sequences^48^ and the Nflnc sequences were used to polish the ICE consensus sequences by Arrow software (Fig. 1). The consensus sequences were further rectified by LoRDEC^49^ software with Illumina RNA-Seq data (Fig. 1).

### PacBio data genome mapping and gene structure analysis

To further improve gene structure annotation, these 300,733 consensus sequences were mapped onto the *Wakin* goldfish reference genome^10^ (https://www.ncbi.nlm.nih.gov/assembly/GCF_003368295.1/) using GMAP^50^ v2017-06–20 with the following parameters: –no-chimeras –cross-species –expand-offsets 1 -B 5 -K 50,000 -f samse -n 1.

Gene structure analysis was performed using TAPIS v1.2.1 pipeline^15^. The GMAP output bam format file and GFF format genome annotation file were used for gene and isoform determination. The high-quality goldfish reference genome contains roughly 70,324 gene models and each gene model contains one transcript isoform^10^. The novel transcript isoforms which possessed different structures from those predicted in the reference genome were identified with the following criteria: 1) the novel transcript isoform contained different exon numbers compared to the reference transcript isoform, or 2) the novel transcript contained the same exon numbers as the reference transcript, but with at least 6-nt difference in the stop coordinate for the first exon, 6-nt difference in the start coordinate for the last exon, or 6-nt difference in the start or stop coordinate for the middle exons.

The new genes were identified by comparing to the reference genes with the following criteria: (1) the query contained no overlap or less than 20% overlapped sequences of the reference gene, or (2) the query contained more than 20% overlapped sequences of the reference gene, but the direction for transcription was different. The related results presented in Fig. 2 were drawn with ggplot 2 R package^51^.

### Alternative splicing and splice isoforms

Alternative splicing (AS) events were explored in goldfish using SUPPA pipeline^52^. Alternative splicing events were divided into seven types: skipping exon (SE), mutually exclusive exons (MX), alternative 5′ splice sites (A5), alternative 3′ splice sites (A3), retained intron (RI), alternative first exon (AF) and alternative last exons (AL).

### Annotation analysis of new transcripts and genes

The unmapped transcripts and new genes mapped onto reference genome were annotated with seven databases including NR, NT, Pfam, KOG, SwissProt, KEGG^53^ and GO databases.

### LncRNA identification and characterization

Long non-coding RNAs (lncRNAs) were identified with CNCI (Coding-Non-Coding-Index), PLEK, CPC (Coding Potential Calculator), and Pfam (Fig. 4a). Briefly, the protein-coding potential of the transcripts were predicted by CNCI and PLEK using the default parameters and blasted in NCBI eukaryotes’ protein database using CPC and Pfam. The parameter for CPC was set at e-value of ‘1e-10′. Pfam hmmscan searching was performed with default parameters of -E 0.001 –domE 0.001. Transcripts with protein-coding potential by one or all aforementioned tools were filtered out, and those without protein-coding potential were candidates for lncRNAs. The related results presented in Fig. 4 were drawn with ggplot 2 R package^51^.

### Global tissue transcriptomic analysis

The Illumina paired-end clean reads were mapped onto the goldfish reference genome using Hisat2 software^54^. The expression level of each isoform was estimated by Cuffdiff v2.1.1^55^, using the updated GFF file containing 87,946 genes from this study and the reference genome. The read numbers of different isoforms for each gene were counted and the gene expression level in FPKM (expected number of Fragments Per Kilobase of transcript sequence per Million base pairs sequenced) was calculated with HTSeq v0.9.1^56^. Gene expression cluster analysis was performed using pheatmap R package 1.0.12 with hierarchical clustering method of Euclidean distance^57^.

### Skin- and color-specific transcriptomic analysis

To identify genes significantly expressed in skin tissue, differentially expressed genes (DEGs) were compared between skin and other tissues with DEGseq R package (1.20.0)^23,58^. The resulting *p*-values were adjusted using the Benjamini & Hochberg’s method to control the false discovery rate (FDR). Gene Ontology (GO) enrichment analysis of DEGs was performed via the GOseq R package of Bioconductor 3.5^59^. GO terms with FDR < 0.05 were identified to be significantly enriched for DEGs. The statistical enrichment (FDR ≤ 0.05) of Kyoto Encyclopedia of Genes and Genomes (KEGG) pathways^53^ for DEGs was determined by KOBAS 3.0 software^60^.

To further identify the genes and pathways related to color-specific skin, we investigated gene expression profiles of four different color skins (black, cyan, red, and white) with RNA-Seq (Fig. 1). DEGs (FDR < 0.05) were compared between any two color skins with DEseq R package (1.18.0)^61^. GO enrichment and KEGG pathway analysis were also performed. The related results were drawn with ggplot 2 R package^51^.

### Comparison of ohnolog genes located in two subgenomes

The gene expression FPKM is the sum of all isoform FPKM of the gene. Log2 FPKM are used for all statistic tests. Pairwise t test was performed using R stats package^62^ with ‘paired = T’. Entropy was computed using entropy R package version 1.2.1^63^. The isoform (or tissue) specificity for each gene is defined as $\mathop {\max }\limits_{i} x_{i}^{2} /\mathop \sum \limits_{i} x_{i}^{2}$, where ${\text{x}}_{{\text{i}}}$ is the expression FPKM of isoform (or tissue) *i*^64^. The related results were drawn with ggplot 2 R package^51^.

### Ethics approval

The animal experiments and all procedures involved in the handling and treatment of goldfish in this study were approved by Shanghai Ocean University. All procedures conducted on fish were performed in accordance with relevant guidelines and regulations. All efforts were made to minimize animal suffering.

## Supplementary information

Supplementary Information.

## Data Availability

The PacBio and Illumina data are publicly available at the NCBI/GenBank database under Bioproject PRJNA587054 and PRJNA580146, respectively. All raw data have been deposited in the NCBI Sequence Read Archive under the accession number SRR10382503-SRR10382505 (PacBio reads) and SRR10358004-SRR10358025 (Illumina reads). The updated GFF file of gene models, the nucleotide and protein sequences of unmapped transcripts, novel-protein-coding genes, and lncRNA genes and other related files were also deposited in Figshare (https://figshare.com/s/dc583090d88df61a640f)^65^.

## References

[1] Wang, C. Y. *Variation and Heredity of Goldfish* (In Chinese). China Agriculture Press (2007).

[2] Ota KG, Abe G (2016). Goldfish morphology as a model for evolutionary developmental biology. Wiley Interdiscip. Rev. Dev. Biol..

[3] Omori Y, Kon T (2019). Goldfish: an old and new model system to study vertebrate development, evolution and human disease. J. Biochem..

[4] Leclercq E, Taylor JF, Migaud H (2009). Morphological skin colour changes in teleosts. Fish Fish..

[5] Braasch I, Schartl M, Volff JN (2007). Evolution of pigment synthesis pathways by gene and genome duplication in fish. BMC Evol. Biol..

[6] Quigley IK (2004). Pigment pattern evolution by differential deployment of neural crest and post-embryonic melanophore lineages in Danio fishes. Development.

[7] Mellgren EM, Johnson SL (2002). The evolution of morphological complexity in zebrafish stripes. Trends Genet..

[8] Braasch I, Brunet F, Volff JN, Schartl M (2009). Pigmentation pathway evolution after whole-genome duplication in fish. Genome Biol. Evol..

[9] Xu P (2014). Genome sequence and genetic diversity of the common carp *Cyprinus carpio*. Nat. Genet..

[10] Chen Z (2019). *De novo* assembly of the goldfish (*Carassius auratus*) genome and the evolution of genes after whole-genome duplication. Sci Adv..

[11] Roberts RJ, Carneiro MO, Schatz MC (2013). The advantages of SMRT sequencing. Genome Biol..

[12] Sharon D, Tilgner H, Grubert F, Snyder M (2013). A single-molecule long-read survey of the human transcriptome. Nat. Biotechnol..

[13] Nudelman G (2018). High resolution annotation of zebrafish transcriptome using long-read sequencing. Genome Res..

[14] Zhang G (2019). PacBio full-length cDNA sequencing integrated with RNA-Seq reads drastically improves the discovery of splicing transcripts in rice. Plant J..

[15] Abdel-Ghany SE (2016). A survey of the sorghum transcriptome using single-molecule long reads. Nat. Commun..

[16] Feng X, Jia Y, Zhu R, Chen K, Chen Y (2019). Characterization and analysis of the transcriptome in *Gymnocypris selincuoensis* on the Qinghai-Tibetan Plateau using single-molecule long-read sequencing and RNA-Seq. DNA Res..

[17] Yi S, Zhou X, Li J, Zhang M, Luo S (2018). Full-length transcriptome of *Misgurnus anguillicaudatus* provides insights into evolution of genus Misgurnus. Sci. Rep..

[18] Teplova M, Patel DJ (2008). Structural insights into RNA recognition by the alternative-splicing regulator muscleblind-like MBNL1. Nat. Struct. Mol. Biol..

[19] Fernandez-Costa JM, Llamusi MB, Garcia-Lopez A, Artero R (2011). Alternative splicing regulation by Muscleblind proteins: from development to disease. Biol. Rev. Camb. Philos. Soc..

[20] Lorin T, Brunet FG, Laudet V, Volff JN (2018). Teleost fish-specific preferential retention of pigmentation gene-containing families after whole genome duplications in vertebrates. G3.

[21] Kimura T (2014). Leucophores are similar to xanthophores in their specification and differentiation processes in medaka. Proc. Natl. Acad. Sci. U. S. A..

[22] Rhoads A, Au KF (2015). PacBio sequencing and its applications. Genom. Proteom. Bioinform..

[23] Da Fonte DF (2017). Secretoneurin A regulates neurogenic and inflammatory transcriptional networks in goldfish (*Carassius auratus*) radial glia. Sci. Rep..

[24] Salem M (2015). Transcriptome assembly, gene annotation and tissue gene expression atlas of the rainbow trout. PLoS ONE.

[25] Zhang R (2015). Local adaptation of *Gymnocypris przewalskii* (Cyprinidae) on the Tibetan Plateau. Sci Rep..

[26] Li J (2017). Long read reference genome-free reconstruction of a full-length transcriptome from *Astragalus membranaceus* reveals transcript variants involved in bioactive compound biosynthesis. Cell Discov..

[27] Zhang X (2019). Full-length transcriptome analysis of *Litopenaeus vannamei* reveals transcript variants involved in the innate immune system. Fish Shellfish Immunol..

[28] Waterhouse RM (2018). BUSCO applications from quality assessments to gene prediction and phylogenomics. Mol. Biol. Evol..

[29] Chen SC (1934). The inheritance of blue and brown colours in the goldfish, *Carassius auratus*. J. Genet..

[30] Kon T (2020). The genetic basis of morphological diversity in domesticated goldfish. Curr. Biol..

[31] Chen Y (2020). Transcriptome analysis identifies candidate genes associated with skin color variation in *Triplophysa siluroides*. Comp. Biochem. Physiol. Part D Genom. Proteom..

[32] Luo M (2019). Integrated analysis of long non-coding RNA and mRNA expression in different colored skin of koi carp. BMC Genomics.

[33] Zhang Y (2017). Comparative transcriptome analysis of molecular mechanism underlying gray-to-red body color formation in red crucian carp (*Carassius auratus*, red var.). Fish Physiol. Biochem..

[34] Mizusawa K, Yamamura Y, Kasagi S, Cerda-Reverter JM, Takahashi A (2018). Expression of genes for melanotropic peptides and their receptors for morphological color change in goldfish *Carassius auratus*. Gen. Comp. Endocrinol..

[35] Zhang Y (2017). Comparative transcriptome and DNA methylation analyses of the molecular mechanisms underlying skin color variations in Crucian carp (*Carassius carassius* L.). BMC Genet..

[36] Curran K (2010). Interplay between Foxd3 and Mitf regulates cell fate plasticity in the zebrafish neural crest. Dev. Biol..

[37] Altschmied J (2002). Subfunctionalization of duplicate *mitf* genes associated with differential degeneration of alternative exons in fish. Genetics.

[38] Cal L, Suarez-Bregua P, Cerda-Reverter JM, Braasch I, Rotllant J (2017). Fish pigmentation and the melanocortin system. Comp. Biochem. Physiol. A Mol. Integr. Physiol..

[39] Kobayashi Y (2011). Pigment-dispersing activities and cortisol-releasing activities of melanocortins and their receptors in xanthophores and head kidneys of the goldfish *Carassius auratus*. Gen. Comp. Endocrinol..

[40] Xue J (2017). The Nrf2/GCH1/BH4 axis ameliorates radiation-induced skin injury by modulating the ROS cascade. J. Invest. Dermatol..

[41] Ziegler I (2003). The pteridine pathway in zebrafish: regulation and specification during the determination of neural crest cell-fate. Pigment Cell Res..

[42] Zhu Z (2020). The comprehensive detection of miRNA, lncRNA, and circRNA in regulation of mouse melanocyte and skin development. Biol. Res..

[43] Ohno S, Muramoto J, Christian L, Atkin NB (1967). Diploid-tetraploid relationship among old-world members of the fish family Cyprinidae. Chromosoma.

[44] Cheng F (2018). Gene retention, fractionation and subgenome differences in polyploid plants. Nat. Plants..

[45] Hu G, Wendel JF (2019). Cis-trans controls and regulatory novelty accompanying allopolyploidization. New Phytol..

[46] Shah M (2020). De novo transcriptome analysis of *Lantana camara* L. revealed candidate genes involved in phenylpropanoid biosynthesis pathway. Sci. Rep..

[47] Hua X (2020). Multi-level transcriptome sequencing identifies COL1A1 as a candidate marker in human heart failure progression. BMC Med..

[48] Eid J (2009). Real-time DNA sequencing from single polymerase molecules. Science.

[49] Salmela L, Rivals E (2014). LoRDEC: accurate and efficient long read error correction. Bioinformatics.

[50] Wu TD, Watanabe CK (2005). GMAP: a genomic mapping and alignment program for mRNA and EST sequences. Bioinformatics.

[51] 51H, W. ggplot2: Elegant graphics for data analysis. Springer-Verlag New York. ISBN 978-3-319-24277-4*,* https://ggplot2.tidyverse.org (2016).

[52] Alamancos GP, Pages A, Trincado JL, Bellora N, Eyras E (2015). Leveraging transcript quantification for fast computation of alternative splicing profiles. RNA.

[53] Kanehisa M, Goto S (2000). KEGG: kyoto encyclopedia of genes and genomes. Nucleic Acids Res..

[54] Kim D, Langmead B, Salzberg SL (2015). HISAT: a fast spliced aligner with low memory requirements. Nat. Methods.

[55] Ghosh, S. & Chan, C. K. Analysis of RNA-Seq data using TopHat and Cufflinks. In: Edwards D. (eds) Plant Bioinformatics. *Methods Mol Biol.* 1374, 339–361. Humana Press, New York, NY. https://doi.org/10.1007/978-1-4939-3167-5_18 2 (2016). 10.1007/978-1-4939-3167-5_18 26519415

[56] Anders S, Pyl PT, Huber W (2015). HTSeq—a Python framework to work with high-throughput sequencing data. Bioinformatics.

[57] Kolde, R. pheatmap: Pretty Heatmaps. R package version 1.0.12. https://CRAN.R-project.org/package=pheatmap (2019).

[58] Wang L, Feng Z, Wang X, Wang X, Zhang X (2010). DEGseq: an R package for identifying differentially expressed genes from RNA-Seq data. Bioinformatics.

[59] Young MD, Wakefield MJ, Smyth GK, Oshlack A (2010). Gene ontology analysis for RNA-Seq: accounting for selection bias. Genome Biol..

[60] Xie C (2011). KOBAS 2.0: a web server for annotation and identification of enriched pathways and diseases. Nucleic Acids Res..

[61] Anders S, Huber W (2010). Differential expression analysis for sequence count data. Genome Biol..

[62] Team, R. C. R: A language and environment for statistical computing. R Foundation for Statistical Computing, Vienna, Austria. URL https://www.R-project.org/ (2020).

[63] Hausser, J. & Strimmer, K. entropy: Estimation of entropy, mutual information and related quantities. R package version 1.2.1. https://CRAN.R-project.org/package=entropy (2014).

[64] Kryuchkova-Mostacci N, Robinson-Rechavi M (2017). A benchmark of gene expression tissue-specificity metrics. Brief Bioinform..

[65] Gan, W. *et al.* Global tissue transcriptomic analysis to improve genome annotation and unravel skin pigmentation in goldfish. *figshare* https://figshare.com/s/dc583090d88df61a640f (2020). 10.1038/s41598-020-80168-6 PMC7815744 33469041

