# Supplementary material for: Global tissue transcriptomic analysis to improve genome annotation and unravel skin pigmentation in goldfish
Source: Sci Rep. 2021 Jan 19;11:1815. doi: 10.1038/s41598-020-80168-6 (PMC7815744; doi:10.1038/s41598-020-80168-6)

Supplementary to manuscript: Global tissue transcriptomic analysis to improve genome annotation and unravel skin pigmentation in goldfish

Wu Gan^1^, Yu-Wen Chung-Davidson^2^, Zelin Chen^3^, Shiying Song^1^, Wenyao Cui^1^, Wei He^1^, Qinghua Zhang^1,4^, Weiming Li^2^, Mingyou Li^1*^, Jianfeng Ren^1,4*^

^1^Key Laboratory of Exploration and Utilization of Aquatic Genetic Resources, Ministry of Education, Shanghai Ocean University, Shanghai 201306, China

^2^Department of Fisheries and Wildlife, Michigan State University, East Lansing, MI 48824, USA

^3^South China Sea Institute of Oceanology, Chinese Academy of Sciences, Guangzhou, 510301, China

^4^International Research Center for Marine Biosciences, Ministry of Science and Technology, Shanghai Ocean University, Shanghai 201306, China

***Correspondence address:** Jianfeng Ren, Shanghai Ocean University, Shanghai 201306, China. E-mail: [jfren@shou.edu.cn](mailto:jfren@shou.edu.cn); Mingyou Li, Shanghai Ocean University, Shanghai 201306, China. E-mail: [myli@shou.edu.cn](mailto:myli@shou.edu.cn).

**Supplementary Fig. S1.** The number of novel genes annotated in seven databases.

**Supplementary Fig. S2.** Gene expression pattern of the newly discovered 11,814 protein-coding genes.

**Supplementary Fig. S3.** Gene expression pattern of 11,742 lncRNA transcripts.

**Supplementary Fig. S4.** BUSCO assessment result of gene completeness of the PacBio Iso-Seq.

**Supplementary Fig. S5.** LncRNA expression across four color skins.

**Supplementary Fig. S6.** The number of differentially expressed lncRNAs among four color skins.

**Supplementary Fig. S7.** The expression pattern of differentially expressed lncRNAs among four color skins.

**Supplementary Fig. S8.** Boxplots of the isoform entropy for all tissues. L-genes have higher isoform entropy than S-genes in most tissues.

**Supplementary Fig. S9.** Boxplots of the isoform specificity for all tissues. L-genes have lower isoform specificity than S-genes in most tissues, but not statistically significant.

**Supplementary Table S1.** Novel isoforms of the reference genes.

See attached Excel file

**Supplementary Table S2.** The statistical information of the novel genes including protein-coding genes and lncRNA genes.

See attached Excel file

**Supplementary Table S3.** Transcript isoform numbers of PacBio Iso-Seq genes.

See attached Excel file

**Supplementary Table S4.** The statistical information of novel gene annotation.

See attached Excel file

**Supplementary Table S5.** Statistical information of alternative spliced genes.

See attached Excel file

**Supplementary Table S6.** Statistical information of all lncRNA genes.

See attached Excel file

**Supplementary Table S7**. Statistical information of Illumina short-read sequencing.

| Sample | SRA Accession No. | Raw Reads | Clean Reads | Clean Bases (Gb) | Mapping Rate (%) |
| --- | --- | --- | --- | --- | --- |
| Bn3 | [SRR10358013](https://dataview.ncbi.nlm.nih.gov/object/SRR10358013) | 54,371,518 | 53,299,664 | 7.99 | 80.45 |
| Ee3 | [SRR10358009](https://dataview.ncbi.nlm.nih.gov/object/SRR10358009) | 58,353,566 | 57,431,302 | 8.61 | 79.73 |
| Fn3 | [SRR10358008](https://dataview.ncbi.nlm.nih.gov/object/SRR10358008) | 43,737,138 | 42,690,862 | 6.40 | 84.44 |
| Gl3 | [SRR10358007](https://dataview.ncbi.nlm.nih.gov/object/SRR10358007) | 44,886,582 | 43,891,722 | 6.58 | 80.14 |
| Hd3 | [SRR10358006](https://dataview.ncbi.nlm.nih.gov/object/SRR10358006) | 47,982,742 | 47,053,884 | 7.06 | 84.31 |
| Ht3 | [SRR10358005](https://dataview.ncbi.nlm.nih.gov/object/SRR10358005) | 39,365,136 | 38,334,340 | 5.75 | 83.14 |
| Ie3 | [SRR10358004](https://dataview.ncbi.nlm.nih.gov/object/SRR10358004) | 46,648,786 | 45,793,952 | 6.87 | 82.74 |
| Me3 | [SRR10358023](https://dataview.ncbi.nlm.nih.gov/object/SRR10358023) | 46,864,850 | 45,878,542 | 6.88 | 86.39 |
| Ky3 | [SRR10358022](https://dataview.ncbi.nlm.nih.gov/object/SRR10358022) | 47,937,208 | 47,184,548 | 7.08 | 83.50 |
| Lr3 | [SRR10358021](https://dataview.ncbi.nlm.nih.gov/object/SRR10358021) | 46,395,252 | 45,018,128 | 6.75 | 83.97 |
| Ts3 | [SRR10358019](https://dataview.ncbi.nlm.nih.gov/object/SRR10358019) | 42,996,116 | 41,997,386 | 6.30 | 83.97 |
| Sp3 | [SRR10358017](https://dataview.ncbi.nlm.nih.gov/object/SRR10358017) | 48,216,754 | 46,949,588 | 7.04 | 84.42 |
| dph1 | [SRR10358025](https://dataview.ncbi.nlm.nih.gov/object/SRR10358025) | 43,398,238 | 42,560,720 | 6.38 | 86.56 |
| dph3 | [SRR10358010](https://dataview.ncbi.nlm.nih.gov/object/SRR10358010) | 47,154,692 | 46,354,324 | 6.95 | 86.34 |
| Sn1 | [SRR10358011](https://dataview.ncbi.nlm.nih.gov/object/SRR10358011) | 67,209,212 | 60,228,650 | 9.03 | 67.09 |
| Sn2 | [SRR10358024](https://dataview.ncbi.nlm.nih.gov/object/SRR10358024) | 45,973,304 | 44,565,894 | 6.68 | 73.55 |
| Sn3 | [SRR10358020](https://dataview.ncbi.nlm.nih.gov/object/SRR10358020) | 46,141,938 | 44,985,302 | 6.75 | 86.51 |
| Sn4 | [SRR10358018](https://dataview.ncbi.nlm.nih.gov/object/SRR10358018) | 45,634,128 | 43,177,898 | 6.48 | 73.44 |
| Sn5 | [SRR10358016](https://dataview.ncbi.nlm.nih.gov/object/SRR10358016) | 43,633,896 | 42,415,670 | 6.36 | 77.47 |
| Sn6 | SRR10358012 | 40,394,402 | 39,797,328 | 5.97 | 83.20 |
| Sn7 | SRR10358015 | 41,003,282 | 40,465,110 | 6.07 | 80.66 |
| Sn8 | [SRR10358014](https://dataview.ncbi.nlm.nih.gov/object/SRR10358014) | 39,577,410 | 38,798,030 | 5.82 | 75.95 |

**Supplementary Table S8.** Differentially expressed genes and pathways in four different color skins (black, cyan, red, and white) of goldfish.

See attached Excel file

**Supplementary Table S9.** Differentially expressed genes involved in pigmentation pathways in different goldfish tissues.

See attached Excel file

**Supplementary Table S10.** The expression levels of the newly discovered protein-coding genes in different goldfish tissues.

See attached Excel file

**Supplementary Table S11.** The expression levels of the newly discovered lncRNA transcripts in different goldfish tissues.

See attached Excel file

**Supplementary Table S12.** Statistical information of PacBio post-filter polymerase.

| Cell ID | Library Size (kb) | Polymerase (bp) | | | | Subreads (bp) | | | |
| --- | --- | --- | --- | --- | --- | --- | --- | --- | --- |
|  |  | Total Bases | Reads Number | Mean Length | N50 Length | Total Bases | Reads Number | Mean Length | N50 Length |
| 1 | 1-2, 2-3, 3-6 | 439,107,931 | 32,146 | 13,660 | 26,250 | 428,611,538 | 211,902 | 2,023 | 2,517 |
| 2 | 1-2, 2-3, 3-6 | 898,399,125 | 65,350 | 13,748 | 27,750 | 878,287,480 | 432,559 | 2,030 | 2,535 |
| 3 | 1-2, 2-3, 3-6 | 337,098,506 | 20,376 | 16,544 | 31,750 | 328,351,698 | 165,120 | 1,989 | 2,469 |
| 4 | 1-2, 2-3, 3-6 | 1,646,221,018 | 76,154 | 21,617 | 39,250 | 1,618,929,723 | 676,847 | 2,392 | 2,903 |
| 5 | 1-2, 2-3, 3-6 | 1,529,721,427 | 93,718 | 16,323 | 34,250 | 1,503,790,456 | 653,380 | 2,302 | 2,847 |
| 6 | 1-2, 2-3, 3-6 | 161,001,071 | 8,156 | 19,740 | 40,750 | 158,255,392 | 68,780 | 2,301 | 2,915 |
| 7 | 1-2, 2-3, 3-6 | 124,848,814 | 5,618 | 22,223 | 39,750 | 122,978,198 | 45,633 | 2,695 | 3,206 |
| 8 | 1-2, 2-3, 3-6 | 135,004,326 | 5,872 | 22,991 | 39,750 | 133,001,775 | 50,350 | 2,642 | 3,153 |
| 9 | 1-2, 2-3, 3-6 | 872,029,196 | 90,801 | 9,604 | 34,750 | 858,447,550 | 383,959 | 2,236 | 2,980 |
| 10 | 0.5-6 | 6,542,552,222 | 446,219 | 14,662 | 33,250 | 6,381,325,758 | 3,220,946 | 1,981 | 2,855 |
| 11 | 0.5-6 | 5,446,236,638 | 229,705 | 23,710 | 42,750 | 5,337,325,300 | 2,347,104 | 2,274 | 3,641 |
| 12 | 0.5-6 | 7,386,436,488 | 305,236 | 24,199 | 43,750 | 7,246,895,656 | 3,191,613 | 2,271 | 3,631 |
| Total | — | 25,518,656,762 | 1,379,351 | 18,500 | — | 24,996,200,524 | 11,448,193 | 2,183 | 3,108 |

**Supplementary Table S13.** The lncRNA expression levels in four different color skins.

See attached Excel file

**Supplementary Table S14.** The expression levels of differentially expressed lncRNAs across four different color skins.

See attached Excel file

**Supplementary Table S15.1.** The isoform number for L, S ohnologs.

**Supplementary Table S15.2.** The averaged FPKM by tissues of all L, S ohnologs.

**Supplementary Table S15.3.** Tissue entropy of all L, S ohnologs.

**Supplementary Table S15.4.** Tissue specificity of all L, S ohnologs.

**Supplementary Table S15.5.** Isoform entropy of all L, S ohnologs in different tissues.

**Supplementary Table S15.6.** Isoform specificity of all L, S ohnologs in different tissues.

See attached Excel file

**Supplementary Fig. S1**

**
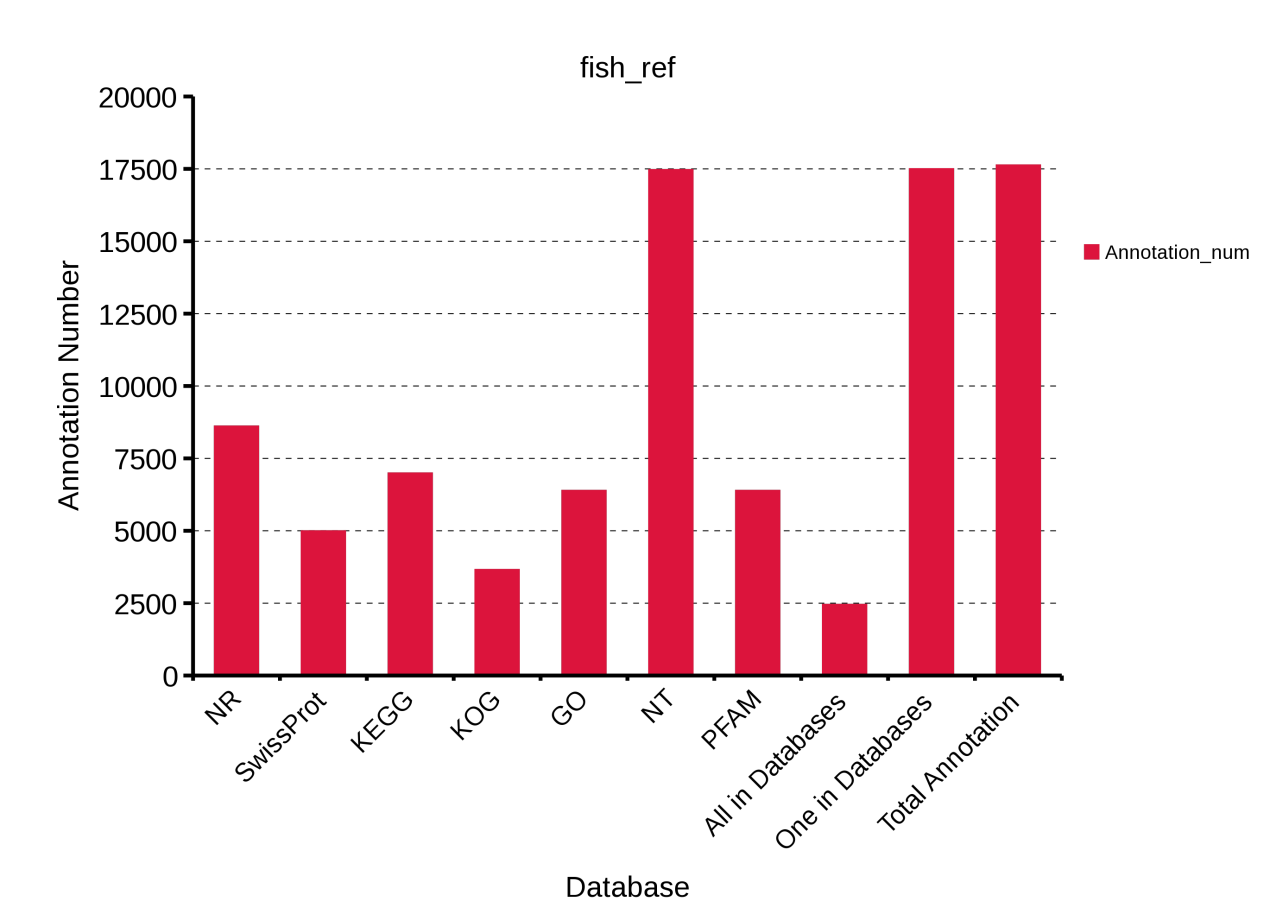
**

**Supplementary Fig. S2**


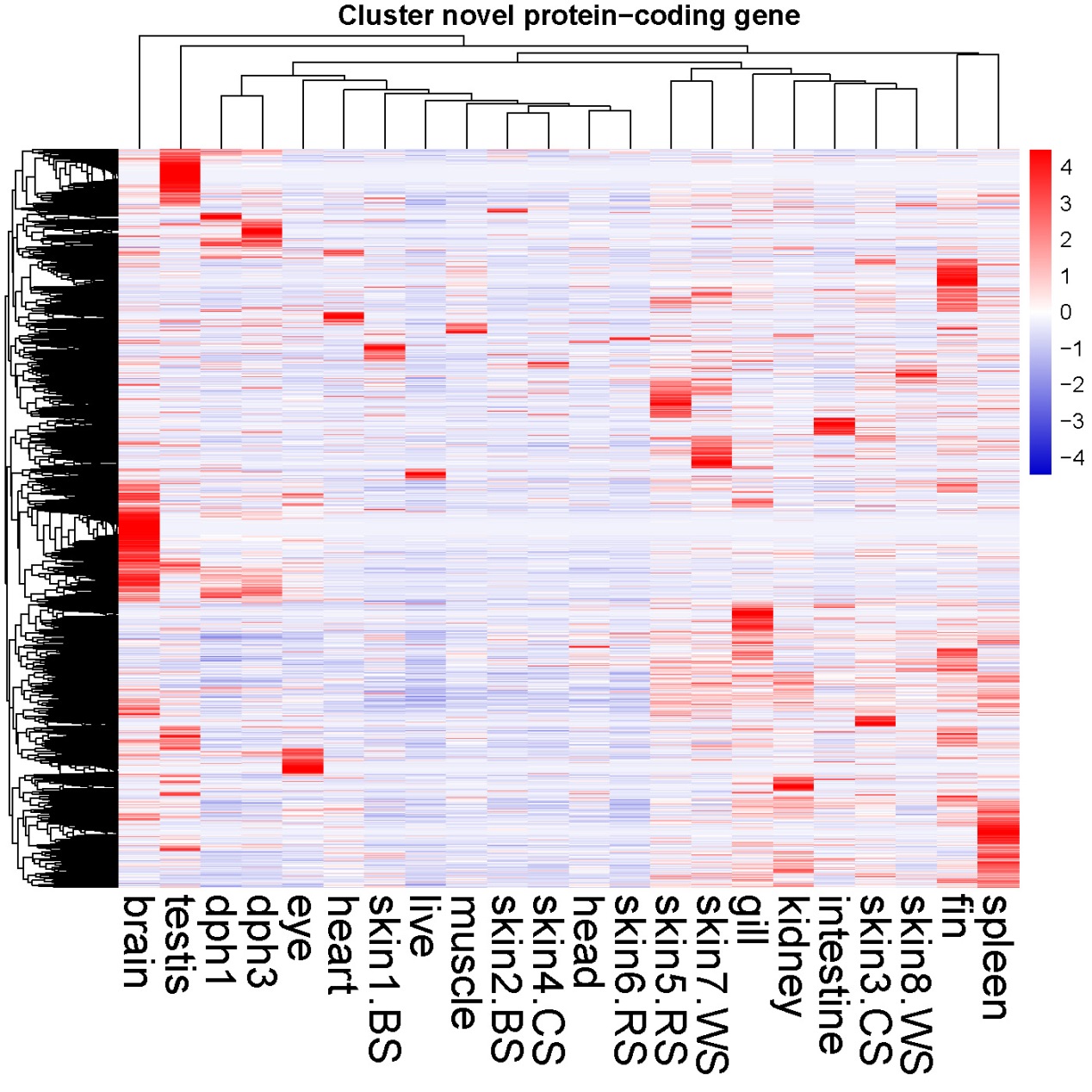


**Supplementary Fig. S3**


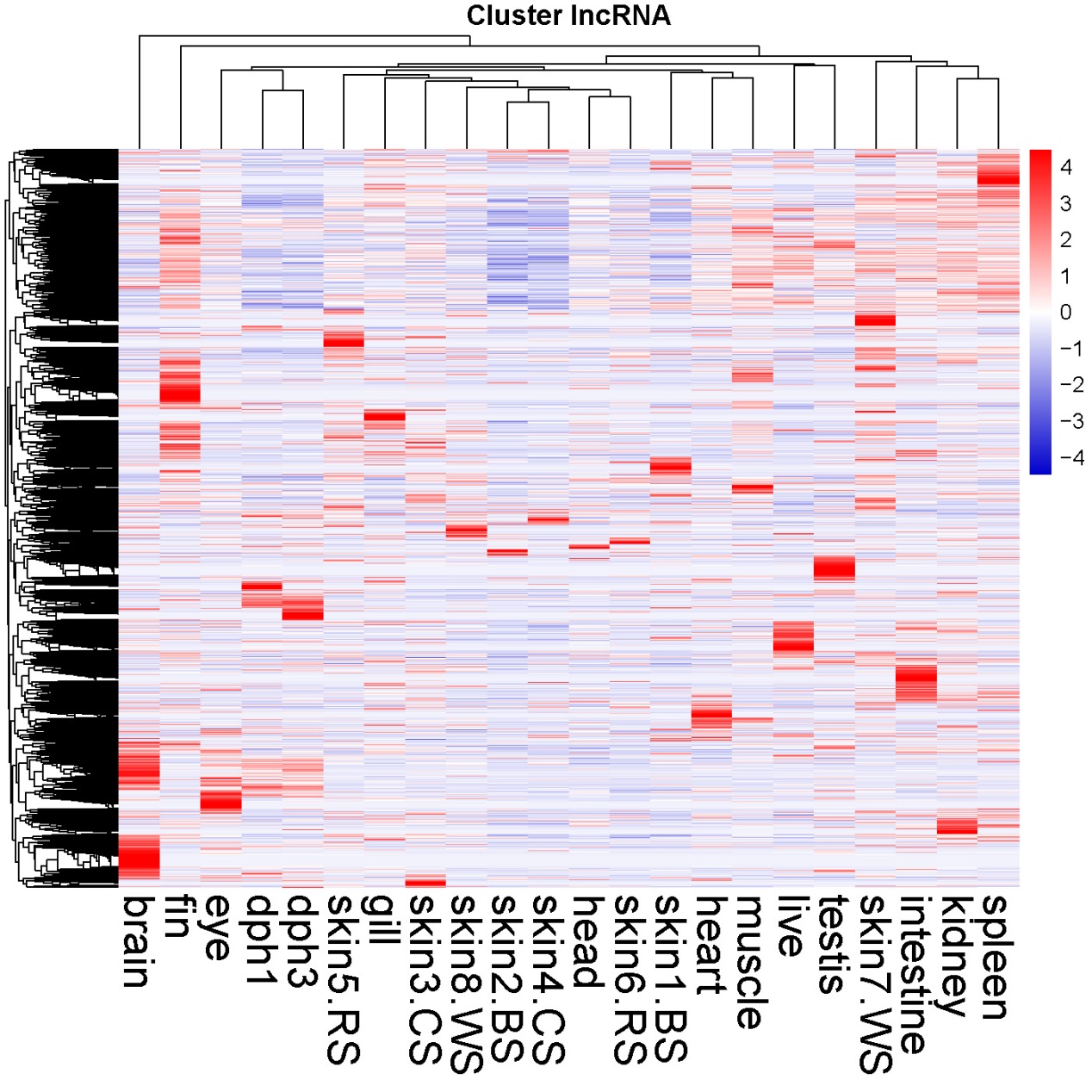


**Supplementary Fig. S4**


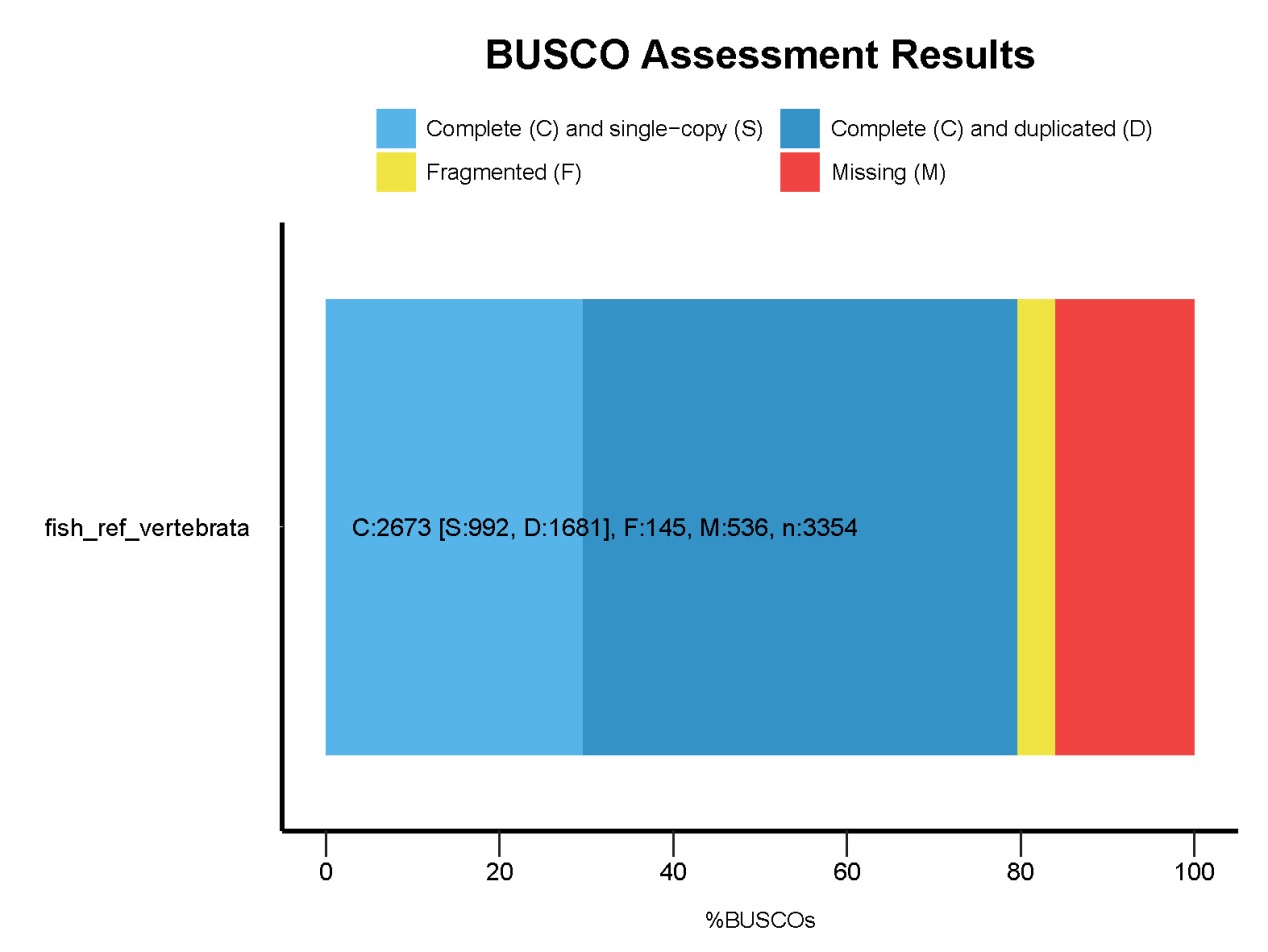


**Supplementary Fig. S5**


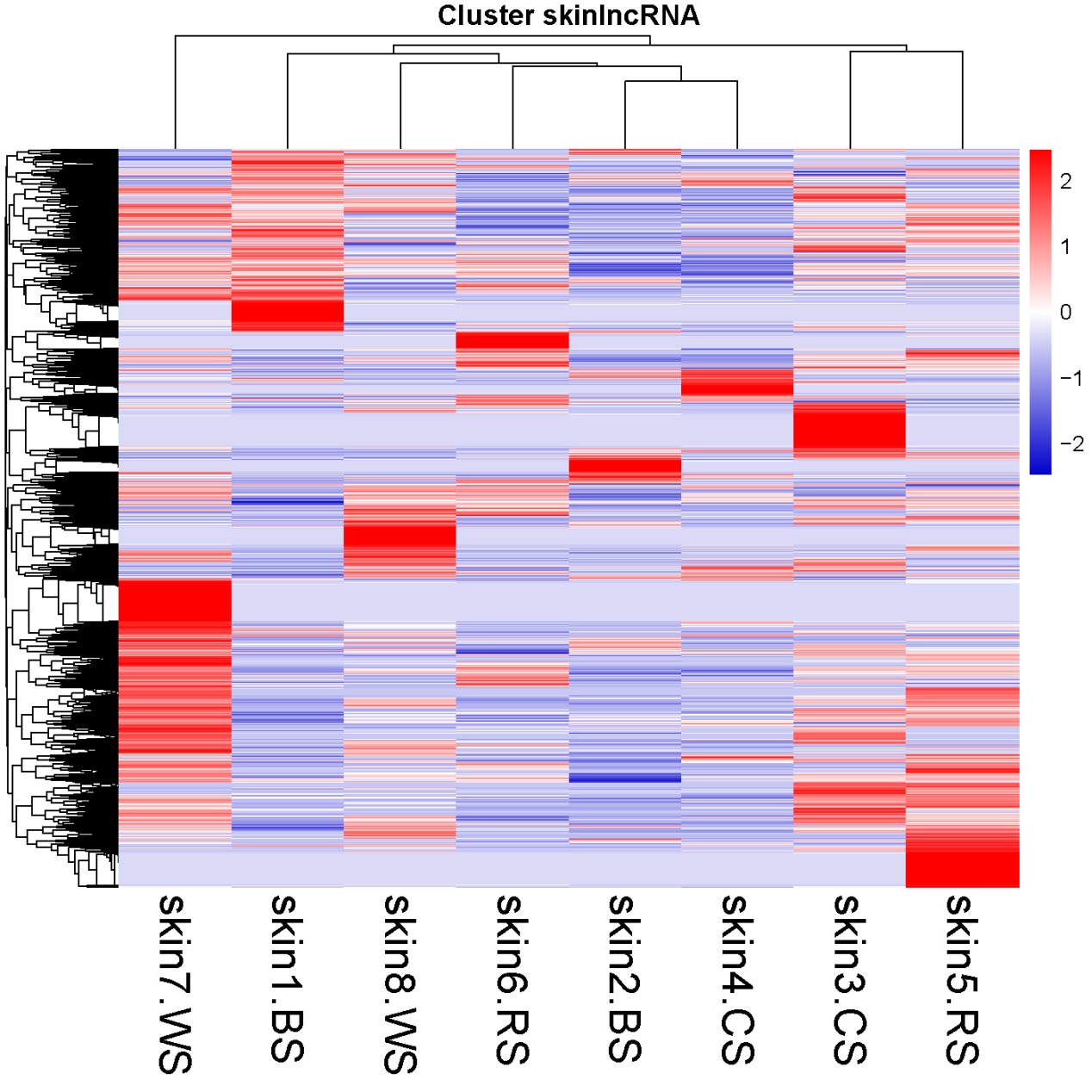


**Supplementary Fig. S6**


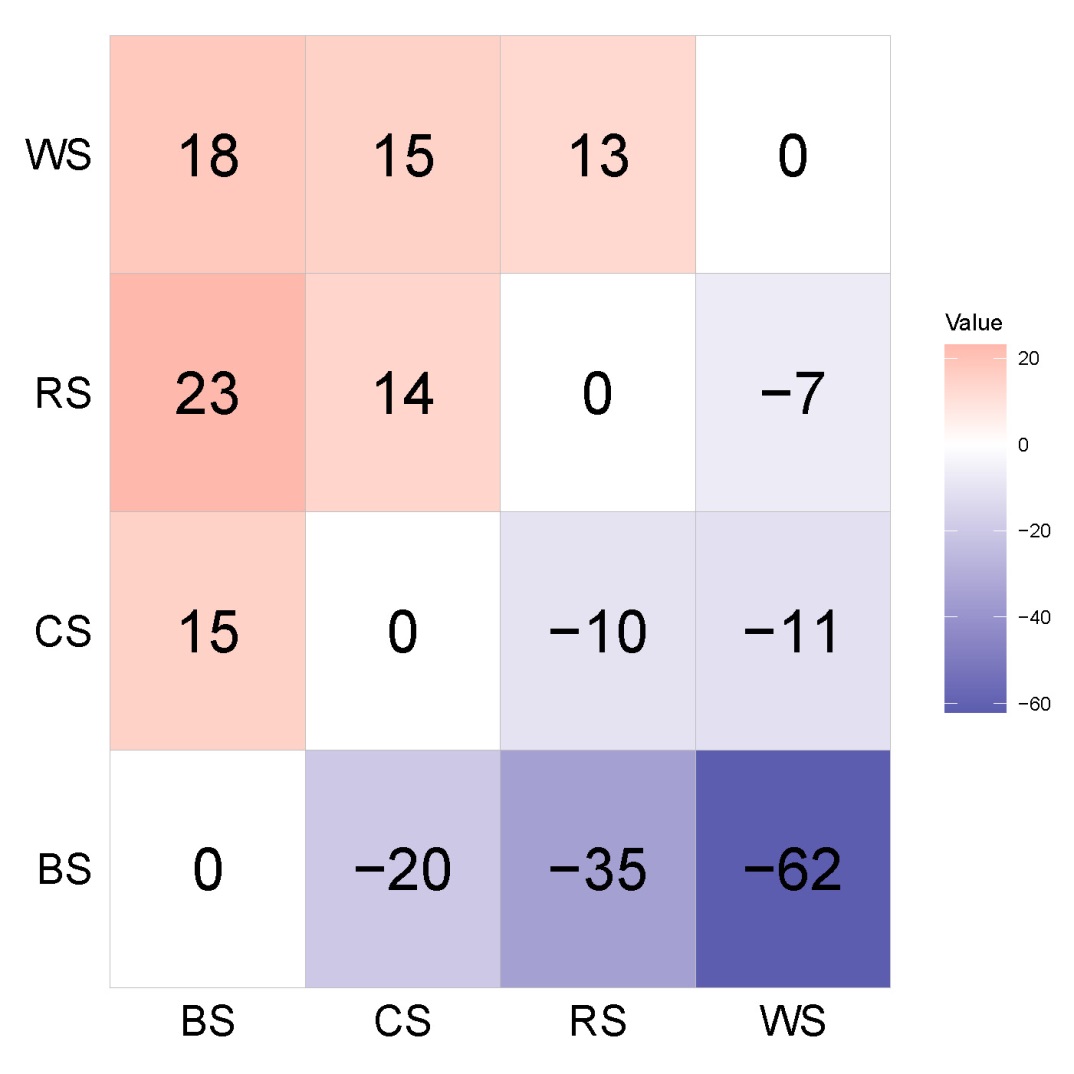


**Supplementary Fig. S7**


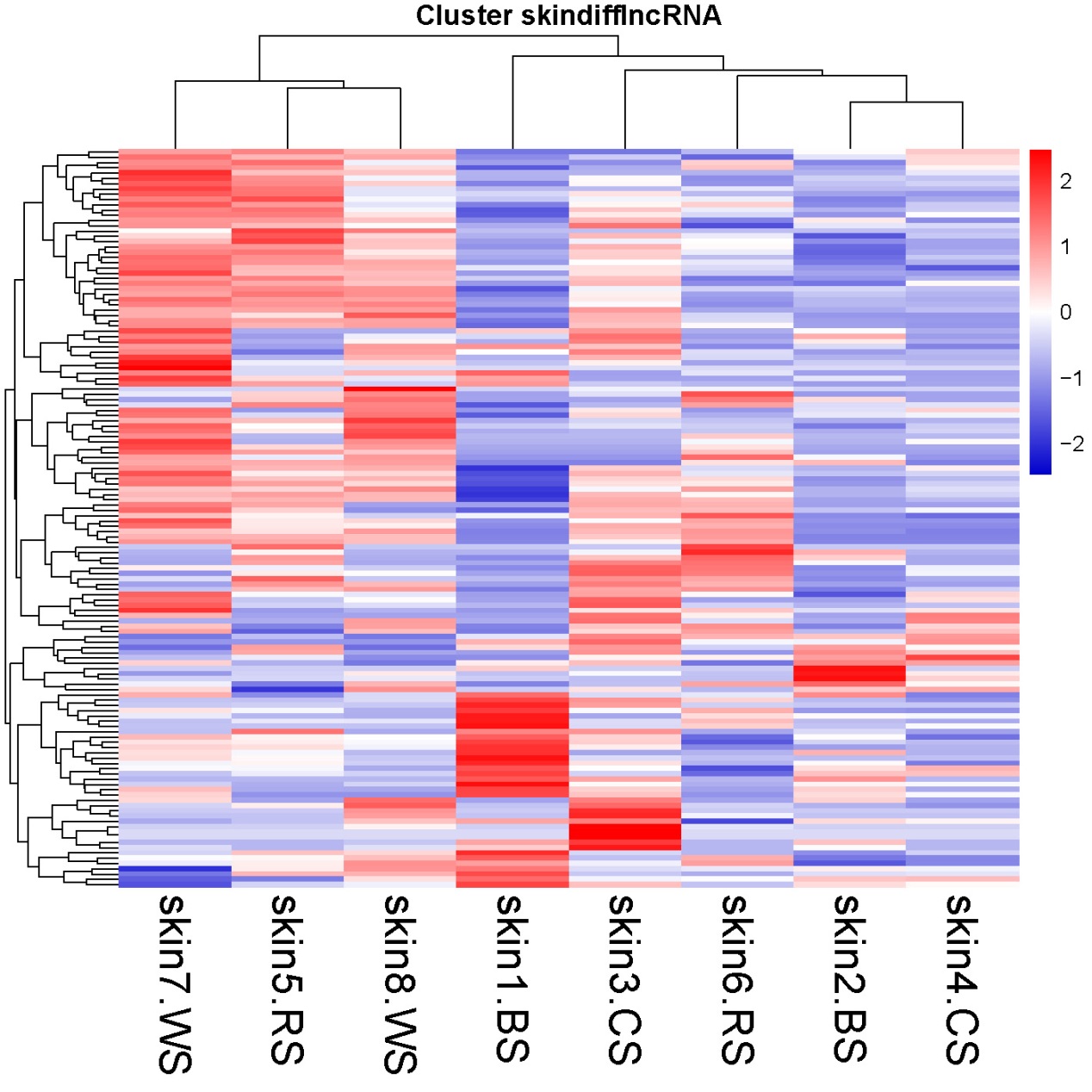


**Supplementary Fig. S8**


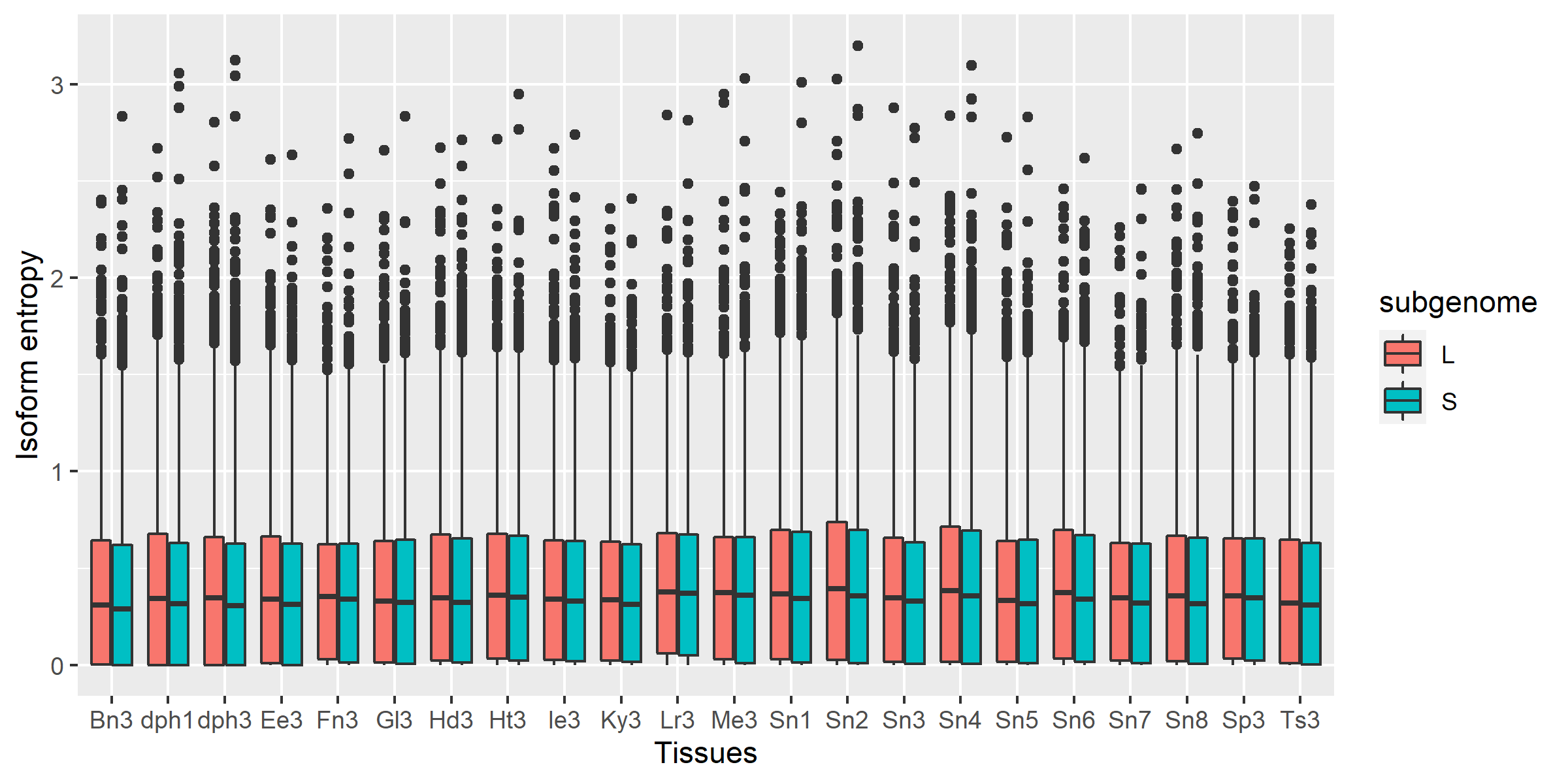


**Supplementary Fig. S9**


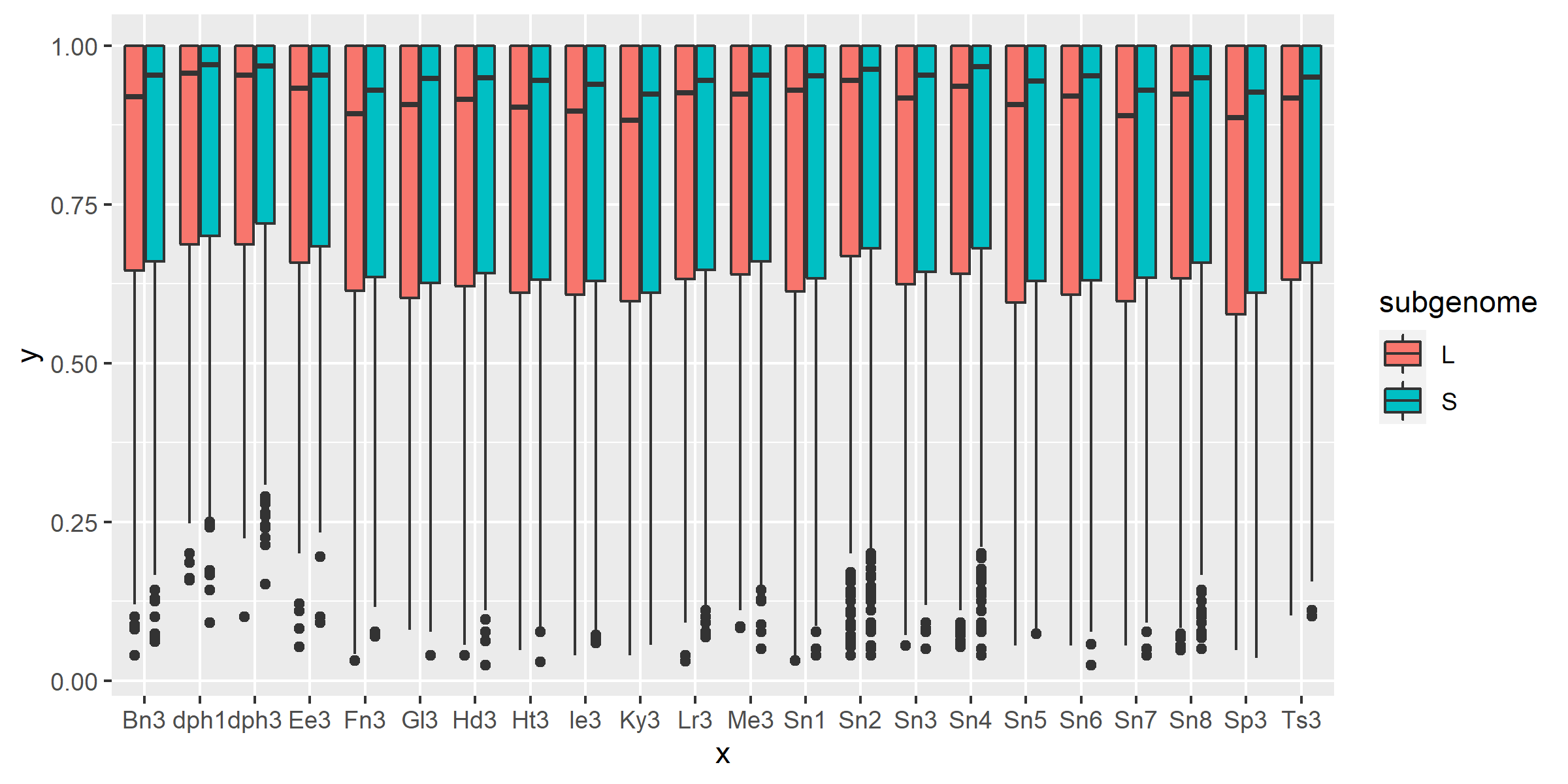

Supplement: Supplementary file 1 — Supplementary Information. [file 41598_2020_80168_MOESM1_ESM.zip › Supplementary Information/Supplementary to manuscript.docx]
